# From sea salt to seawater: a novel approach for the production of water CRMs﻿

**DOI:** 10.1007/s00216-022-04098-0

**Published:** 2022-05-12

**Authors:** Enea Pagliano, Kenny Nadeau, Ovidiu Mihai, Indumathi Pihillagawa Gedara, Zoltán Mester

**Affiliations:** National Research Council Canada, 1200 Montreal Road, Ottawa, ON K1A 0R6 Canada

**Keywords:** Seawater, Phosphate, Silicate, Nitrate, Certified reference materials, Nutrients

## Abstract

Natural water certified reference materials (CRMs) are mostly available in a liquid form, and they are produced starting from suitable environmental samples. Many precautions are usually needed to avoid biological or physical degradation, including filtration, acidification, and sterilization. In this study, the drawbacks associated with liquid-based seawater CRMs were tackled by developing a salt-based seawater proxy for nutrients that could be reconstituted in water solution just before use. Phosphate, silicate, and nitrate were chosen as target analytes. Sea salt mimicking the composition of seawater was spiked with an aqueous solution of the analytes and homogenized using a high-energy planetary ball mill (*u*_hom_ < 1.2%). The salt powder CRM SALT-1 (https://doi.org/10.4224/crm.2022.salt-1) demonstrated good short- and long-term stability for nutrients. When the SALT-1 was reconstituted in water at the 4.0% *w*/*w* level, the resulting solution had similar properties with respect to typical seawater in terms of major constituents (± 20%), trace metals, density (1.023 g/mL), pH (8.8–9.0), and optical properties relevant to the photometric characterization. Phosphate and silicate were quantified by photometry (molybdenum blue method, batch mode), whereas nitrate was quantified by isotope dilution GC−MS (*u*_char_ < 1.2%). In the SALT-1 reconstituted seawater solution at the 4.0% *w*/*w* salt level, the nutrient amount concentration was *w*(phosphate, PO_4_^3−^) = 1.615 ± 0.030 μmol/L, *w*(silicate as SiO_2_) = 8.89 ± 0.31 μmol/L, and *w*(nitrate, NO_3_^−^) = 18.98 ± 0.45 μmol/L at the 95% confidence (*k* = 2). Overall, the SALT-1 CRM exhibits similar nutrient profile and general analytical characteristics as the MOOS-3 CRM. However, the SALT-1 has much reduced preparation, storage, and distribution cost, likely much better long-term stability, and it could enable the production of lower cost and more accessible seawater reference materials.

**Graphical abstract:**

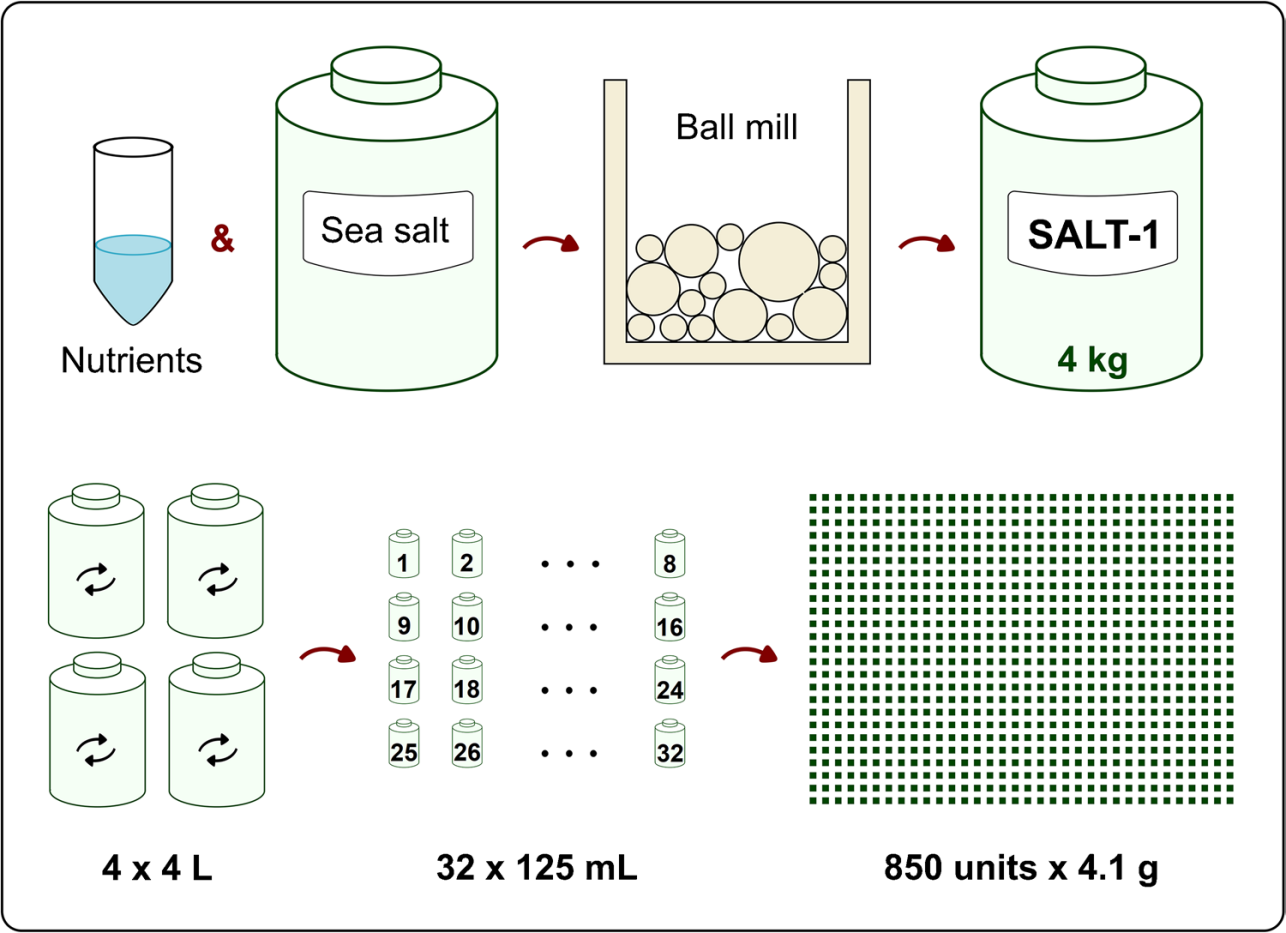

**Supplementary Information:**

The online version contains supplementary material available at 10.1007/s00216-022-04098-0.

## Introduction

Certified reference materials (CRMs) play an essential role in quality control and method validation, particularly for the evaluation of trueness of measurement procedures [1, 2]. Over time, the growth in production and availability of CRMs has gone hand in hand with an increased number of testing laboratories claiming compliance with general competence guidelines like the ISO/IEC 17025 [3, 4]. Some of the latest developments in CRM production can be found in the 2015 special issue *Reference Materials for Chemical Analysis* published by this journal [1] and in two recent reviews [3, 5]. The preparation of natural water CRMs for environmental monitoring of trace metals, organometallics, organic pollutants, and nutrients is a topic of paramount importance for CRM producers. Since the 1980s, the National Research Council Canada (NRC) has been engaged in the preparation of CRMs for quality assurance of water analysis [6], and in the last decade, the institute released a seawater CRM for inorganic nutrients (MOOS-3 [7, 8]), two seawater CRMs for trace metals (NASS-7, CASS-6 [9]), and two CRMs for trace metals in drinking water (AQUA-1 [10]) and in river water (SLRS-6 [11]). Other recent CRM developments in this area include trace metal–spiked costal water produced by the National Measurement Institute Australia (NMIA) [12], the candidate water CRMs for mercury [13] and polycyclic aromatic hydrocarbons (PAHs) [14] by the Joint Research Center (JRC), and the candidate seawater CRM certified for iron by the TÜBİTAK National Metrology Institute [15]. Finally, in the area of nutrient analysis, it is worth mentioning the CRMs from the National Metrology Institute of Japan [16] and the proficiency test samples offered by WEPAL/QUASIMEME (https://www.wepal.nl/en/wepal/Home/Proficiency-tests.htm).

The preparation of water CRMs is a challenging task, particularly for low-level organic contaminants where the stability of the analytes during preparation and long-term storage is known to be an issue [17–19]. This shortcoming resulted in a lack of suitable CRMs for environmental monitoring schemes required by the European Water Framework Directive 2000/60/EC [20]. For this reason, production strategies able to deliver suitable CRM for environmental monitoring of natural water and seawater are the subject of active research [14, 21].

When facing the issues of stability, CRM producers resort to processing methods common in the food industry such as lowering storage temperature and reducing water content of the samples [22–26]. The resulting dry(-er) powders are more inert toward biological activity and can be stored for a longer time. In the late 1990s, lyophilization, as a benign drying process, was also explored for the production of natural water CRMs [17–19]. Most of the efforts were channeled to the preparation of dry materials which could be employed for quality control of polar pesticides in waters. In the pioneering work by Barceló et al. [27–29], several pesticides were spiked in a 150 L-water batch along with 0.6% of glycine as excipient. The solution was freeze-dried, and the pesticide content in the resulting powder was more stable than the aqueous formulation; however, the large concentration of glycine in the reconstituted samples interfered in the chromatographic determination of the pesticides. For this reason, Martín-Esteban et al. [30, 31] replaced glycine with 2.5 g/L NaCl and applied the freeze-dry process for the preparation of proficiency test materials [30] and CRMs [31] for polar pesticides. Similar work was proposed by Vercoutere et al. for the production of a lyophilized CRM solution certified for Cr(III) and Cr(VI) [32].

Although homogeneity of the lyophilized powder was satisfactory for most analytes, some exceptions were reported. For certain molecules, like permethrin, the process caused significant analyte losses and inhomogeneity [30, 31]. Similarly, the stability of the analyte was dependent on its chemical identity and on temperature; however, in most cases, no instability was detected when the material was kept at -20 °C for over a year. This evidence suggests that the production of solid CRMs as a proxy for water analysis could be a viable approach for specific applications. The major drawback of this method was the use of excipients (glycine, NaCl). When the powder was reconstituted in water, composition and pH were not commutable with natural water samples. Furthermore, the lyophilization would have been challenging for seawater, requiring freeze-dry facilities able to process large batches of water without contamination [31].

In this study, a more efficient production scheme for the preparation of water proxy CRMs was explored. This method was applied for the preparation of a sea salt matrix spiked with inorganic nutrients [33–36]. Since seawater-based CRMs for nutrients are difficult to prepare and stabilize [37], the dry format was evaluated as an alternative. A sea salt material (SALT-1 CRM) that could be used to simulate seawater was prepared [38]. For this purpose, an artificial sea salt intended for aquarists was spiked with the analytes of interest directly onto the dry material. Mixing, homogenization, and comminution were obtained with planetary ball milling. The reconstituted sample closely mimic chemical and physical properties of natural seawater samples and the homogeneity and stability of the material were suitable for CRM applications.

## Materials and methods

### Reagents and materials

Kent Marine Reef Salt (Central Aquatics, USA) was used as the matrix for SALT-1 preparation. This commercial formulation did not contain a significant amount of inorganic nutrients; therefore, it was spiked using an aqueous solution containing ACS–grade sodium nitrate, sodium phosphate monobasic monohydrate (Sigma-Aldrich), and sodium metasilicate nonahydrate (NIST SRM 3150 silicon standard solution, National Institute of Standards and Technology).

Concentrated hydrochloric acid (*w* = 36.5–38.0%) was acquired from Fisher Scientific. Standard 0.1 M hydrochloric acid was obtained from Acros Organics (Lot. B00U5294). All other reagents (ACS grade) were purchased from MilliporeSigma: sulfanilamide (*w* ≥ 0.99), *N*-(1-naphthyl)ethylenediamine dihydrochloride (*w* ≥ 98%), sulfuric acid concentrate (*w* = 95.5–96.5%), ammonium molybdate tetrahydrate (*w* = 99.98%), potassium antimony(III) tartrate hydrate (*w* = 99.95%), l-ascorbic acid (*w* ≥ 99%), oxalic acid dehydrate (*w* ≥ 99.5%), triethyloxonium tetrafluoroborate (*w* ≥ 97%), sulfamic acid (*w* = 99.999%). Isotopically ^15^N-enriched nitrite and nitrate were obtained from Cambridge Isotope Laboratories: Na^15^NO_3_ (*w*(^15^N) ≥ 98%) and Na^15^NO_2_ (*w*(^15^N) ≥ 98%). High-purity water (exceeding ISO 3696 grade 1 standard) was generated in-house with a Thermo Scientific Gen-Pure UV xCAD plus system (18.2 MΩ cm at 25 °C).

Quantitation of nutrients in SALT-1 (phosphate, silicate, and nitrate) was performed against two sets of standards. The first set was obtained from the National Institute of Standards and Technology: SRM 3186 (Lot. 170606, *w*(PO_4_^3−^) = 1.0005 mg/g ± 0.0041 mg/g, *k* = 2.284), SRM 3150 (Lot. 130912, *w*(Si) = 9.901 mg/g ± 0.023 mg/g, *k* = 2.021), and SRM 3185 (Lot. 170309, *w*(NO_3_^−^) = 1.0006 mg/g ± 0.0018 mg/g, *k* = 1.965). The second set of standards was obtained from MilliporeSigma: P/N 38364 (Lot. BCCB6423, *w*(PO_4_^3−^) = 1000 mg/kg ± 4 mg/kg, *k* = 2), P/N 18895 (Lot. BCCB8928, *w*(SiO_2_) = 997 mg/kg ± 6 mg/kg, *k* = 2), and P/N 74246 (Lot. BCCC1546, *w*(NO_3_^−^) = 1001 mg/kg ± 4 mg/kg, *k* = 2). For quality control, the NRC MOOS-3 CRM was also analyzed [7].

### Instrumentation

Comminution and homogenization of the SALT-1 material was performed using a planetary ball mill (PQ-N4, Across International, 1-L zirconia jars with yttrium-stabilized zirconium oxide milling balls). Further bulk homogenization was carried out using an elliptical shaker (Red Devil). The bottling of the material was performed using a Mettler Toledo Quantos carousel for automatic powder dispensing and the weight of each SALT-1 unit was read on a Mettler Toledo XPE205 balance equipped with an antistatic kit.

The pH of the SALT-1 aqueous solutions was measured using a Mettler Toledo SevenExcellence pH Meter (glass membrane electrode, P/N 51344055; 3-point calibration: pH 4.01, 7.01, 9.21), whereas the density was measured using a 25-mL pycnometer from MilliporeSigma. All operations of sample preparation and dilutions were performed gravimetrically on a Mettler Toledo MS204S balance calibrated against NRC reference masses. A Thermo Scientific Evolution 220 UV–Visible spectrophotometer was used for all measurements of absorbance in batch mode with either 1- or 5-cm Hellma cuvettes made of Suprasil quartz. For nitrate measurements, a 5973 Hewlett-Packard GC–MS system with a CTC CombiPAL autosampler was used in negative chemical ionization mode (methane). As a confirmation method, an HPLC ICP−MS/MS procedure was also implemented for quantitation of phosphate and silicate. For this purpose, an Agilent 1200 series HPLC was coupled with an Agilent 8800 ICP−MS/MS. The screening of major anions and metal constituents was performed using a Thermo Scientific ion chromatography system ICS-5000^+^ and an Agilent 5110 SVDV ICP−OES. For the screening of the trace metals, an Elemental Scientific seaFAST automated preconcentration system was combined with a Thermo Element XR high-resolution ICP−MS. Details about instrumental settings for all methods are provided in the ESM.

### Determination of nutrients

The determination of phosphate and silicate was carried out using the photometric molybdenum blue method [39, 40]. Phosphate was reacted with ammonium molybdate and antimony potassium tartrate in sulfuric acid medium. The resulting phosphomolybdic acid was then reduced by ascorbic acid and the absorbance was measured at 890 nm. Similarly, the silicomolybdic complex was generated by reaction of the sample with ammonium molybdate in acidic solution, followed by reaction with oxalic acid and reduction by ascorbic acid. In this case, the peak of absorbance was located at 810 nm. The nitrate was measured by isotope dilution headspace GC−MS as described previously [41–43]. Briefly, the sample was spiked with ^15^NO_3_^−^ internal standard, reacted with sulfamic acid for nitrite removal and alkylated using triethyloxonium tetrafluoroborate to convert NO_3_^−^ into EtONO_2_, a derivative suitable for static headspace GC−MS analysis [44, 45]. Nitrite was detected using photometry by the diazotization reaction with sulfanilamide and *N*-(1-naphthyl)-ethylenediamine dihydrochloride in acidic medium [39]. Phosphate and silicate were also measured by HPLC ICP−MS/MS [8]. The analytes were separated on an ion exclusion IonPac ICE-AS1 analytical column and measured by ICP−MS/MS in H_2_ mode. Procedural details are described in the ESM (Paragraphs S1.1 to S1.5).

## Results and discussion

The objective of this study was the preparation of a salt-based material which could be reconstituted in water and used as a quality control test sample for the determination of nutrients in seawater. In the following paragraphs, the preparation and the certification of the SALT-1 CRM are described. Besides the assessments required for certification (i.e., homogeneity, stability, and determination of the property values), attention was given to the characterization of the most relevant properties of the 4.0% *w*/*w* SALT-1 aqueous solution, such as density, pH, UV–vis spectra, major components, and trace metals to ensure that the material was an appropriate proxy for natural seawater.

### Preparation of the SALT-1 CRM

**Fig. 1:**
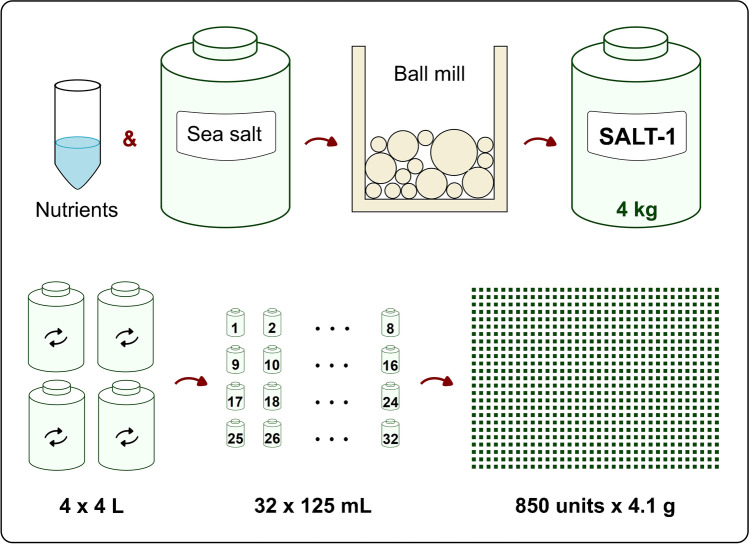
Preparation of the NRC SALT-1 CRM. 1.5 mL of nutrient solution (nitrate, phosphate, and silicate) was transferred in a zirconia jar with 600 g of artificial sea salt and homogenized in a planetary ball mill. Four kilograms of material was recovered in a 20-L carboy, portioned in 4 × 4-L plastic jars, further homogenized in an elliptical shaker, portioned in 32 × 125 mL bottles, and finally bottled into 850 CRM units

A combined nutrient spike solution was prepared in water at these levels of mass fraction: *w*(NO_3_^−^) = 1.15%, *w*(Si) = 0.217%, *w*(PO_4_^3−^) = 0.145%. A 1.5-mL aliquot (1.54 g) of this solution was mixed with 600 g of marine salt in a planetary ball mill. Specifically, 1.5 mL nutrient solution and 600 g marine salt were mixed in a 1-L zirconia jar containing milling zirconia balls (⌀5 mm = 940; ⌀10 mm = 154; ⌀20 mm = 3). The material was milled using a 90-min program where the ball mill was operated at 42 Hz in bidirectional mode: 10 min on and 10 min off. Before opening, the jars were left to cool for 40 min. In a milling cycle, two 1-L jars were employed. The final product (~ 1 kg) was sieved into a 4-L PE bottle, and further homogenized for 15 min using an elliptical shaker. For the production of SALT-1, the process was repeated 4 times yielding ~ 4 kg of product. For further homogenization, the material was transferred into a 20-L PE carboy and shaken manually for 5 min; then, it was portioned back in 4 × 4-L plastic bottles and shaken again for 30 min on the elliptical shaker. The final salt mix was then dispensed into 32 × 125-mL plastic bottles (~ 115 g each) which were thermosealed in trilaminate bags. The material was finally aliquoted into 850 × 12-mL PE bottles (Fig. 1). The target mass of SALT-1 in each unit was 4.1 g, and the mass of each unit was recorded. The mass distribution of the SALT-1 lot is reported in Fig. S1.

### SALT-1 properties

#### Hygroscopicity of the SALT-1

The manipulation of this large surface area (potentially hygroscopic) material posed some initial moisture adsorption concerns. Therefore, its atmospheric moisture adsorption was studied. 3.5 g of SALT-1 was spread on a Petri dish and the mass increase due to moisture adsorption was < 1.5% over 1-h time (*t* = 20 °C, RH = 40–60%, Fig. S2). Since the preparation of each individual SALT-1 unit took ~ 2.1 min (Fig. S3), all manipulations were carried out under normal laboratory conditions and the finished material was kept tightly closed in PE bottles sealed into trilaminated pouches. Under these conditions, the long-term storage was also favorable. Over a period of 3 years, the average mass increase for the SALT-1 units was only 0.28% (*n* = 49, RSD = 38%).

#### Reconstitution: optical properties and pH

The SALT-1 was reconstituted in water to a final mass fraction of 4.0% *w*/*w*. This composition was chosen to match average seawater matrix with a density of 1.023 g/mL. Although SALT-1 solubilized instantaneously, the reconstituted material showed a slight opalescence barely noticeable by naked eye. While studying a possible explanation for this effect, it was noticed that the pH of the SALT-1 solution (pH 8.8–9.0) was at the high end of the ocean water pH interval [46]. Moreover, the pH of the material was influenced by the storage temperature: when the SALT-1 was baked at 105 °C for 21 days, the pH of its 4.0% *w*/*w* solution was 10.5 and it looked quite turbid. Since sodium bicarbonate is a likely component of the artificial sea salt used to prepare the SALT-1, it was hypothesized that the “loss of acidity” caused by heating could be due to thermal degradation of bicarbonates [47]:

$${2\mathrm{NaHCO}}_{3}\left(\mathrm{s}\right)\to {\mathrm{Na}}_{2}{\mathrm{CO}}_{3}\left(\mathrm{s}\right)+{\mathrm{H}}_{2}\mathrm{O}\left(\mathrm{g}\right)+{\mathrm{CO}}_{2}\left(\mathrm{g}\right)$$

Even milder conditions could cause a slight pH increase: a SALT-1 unit left at 40 °C for 21 days generated a solution with a pH of 9.1.

**Fig. 2:**
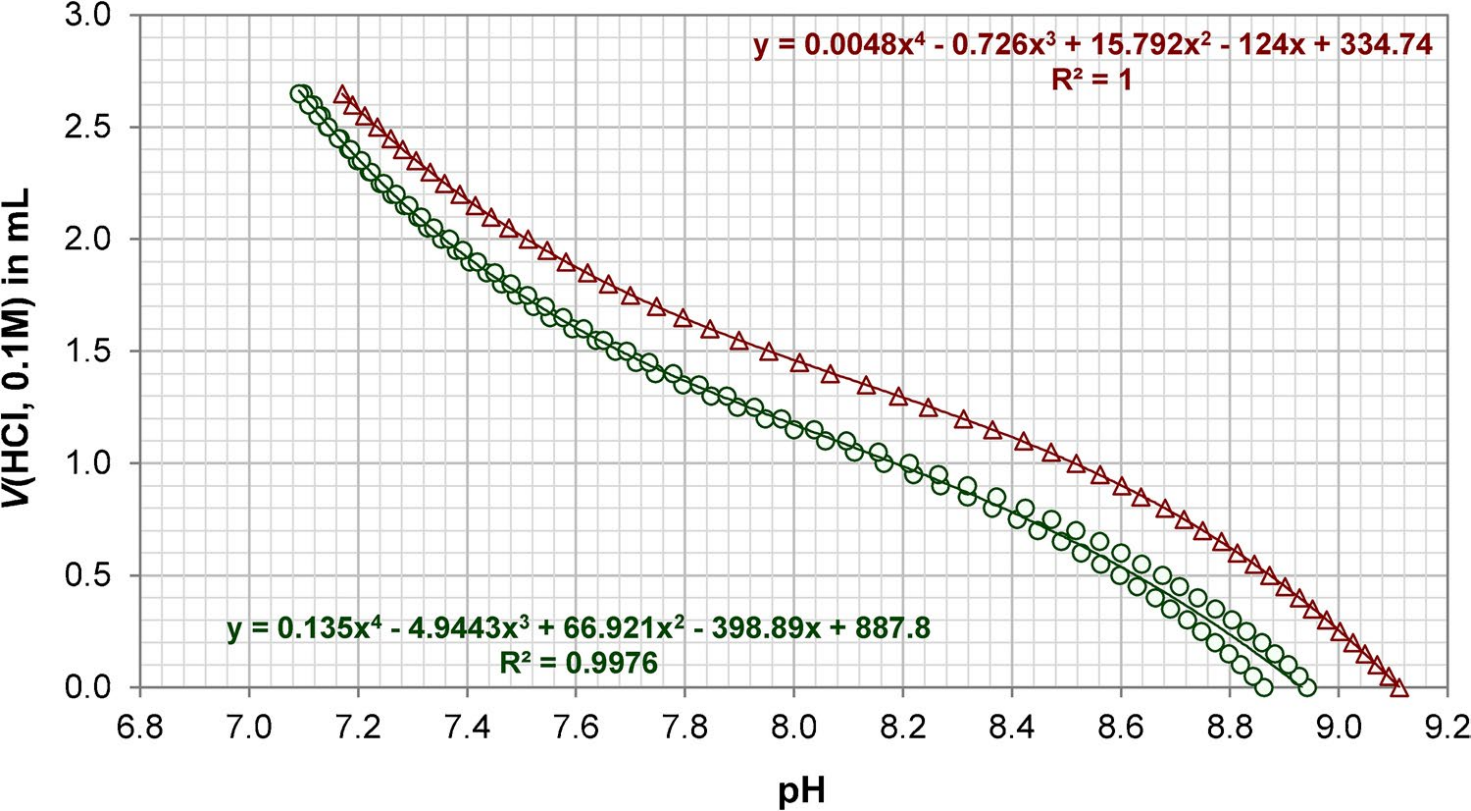
pH of the reconstituted SALT-1 solution (i.e., ~ 4.1 g of SALT-1 in 100 mL volume of solution) after addition of HCl 0.1 M. Green circles: two SALT-1 units kept at 4 °C; red triangles: one SALT-1 unit kept at 40 °C for 21 days. The SALT-1 samples were dissolved in water. The solutions were kept under magnetic stirring and the initial pH was read after 3 min equilibration time. Fifty microliters of 0.1-M HCl aliquots was then added, and the pH was read 30 s after every additions. *y*-axis: volume of 0.1 M HCl added; *x*-axis: pH reading

The alkaline excess could be corrected by adding acid to the SALT-1 solution. Figure 2 reports the pH variations by adding 50-μL aliquots of 0.1 M HCl into the reconstituted solution of SALT-1 (i.e., ~ 4.1 g of SALT-1 in 100 mL volume of solution). When 1.5 mL of 0.1 M HCl was used to correct acidity, the pH of the resulting solution was between 7.7 and 8.0 (a pH between 7.2 and 7.3 could be obtained by adding 2.4 mL of 0.1 M HCl).

The slight opalescence of the SALT-1 solution was related to pH and likely depended upon the complex aqueous equilibria that regulates solubility and precipitation of Mg(OH)_2_ and CaCO_3_ [48]. When the SALT-1 solution was buffered to pH values lower than 8.2, the opalescence disappears and the residual absorbance of the matrix was comparable to the values obtained in low-nutrient seawater (Fig. S4). Furthermore, under acidic conditions typically required for the determination of phosphate, silicate, and nitrate by photometry [39], the residual absorption of the matrix was comparable to low-nutrient seawater, even when the SALT-1 was baked at 105 °C (Fig. S5).

#### Reconstitution: nutrient solubilization

**Fig. 3:**
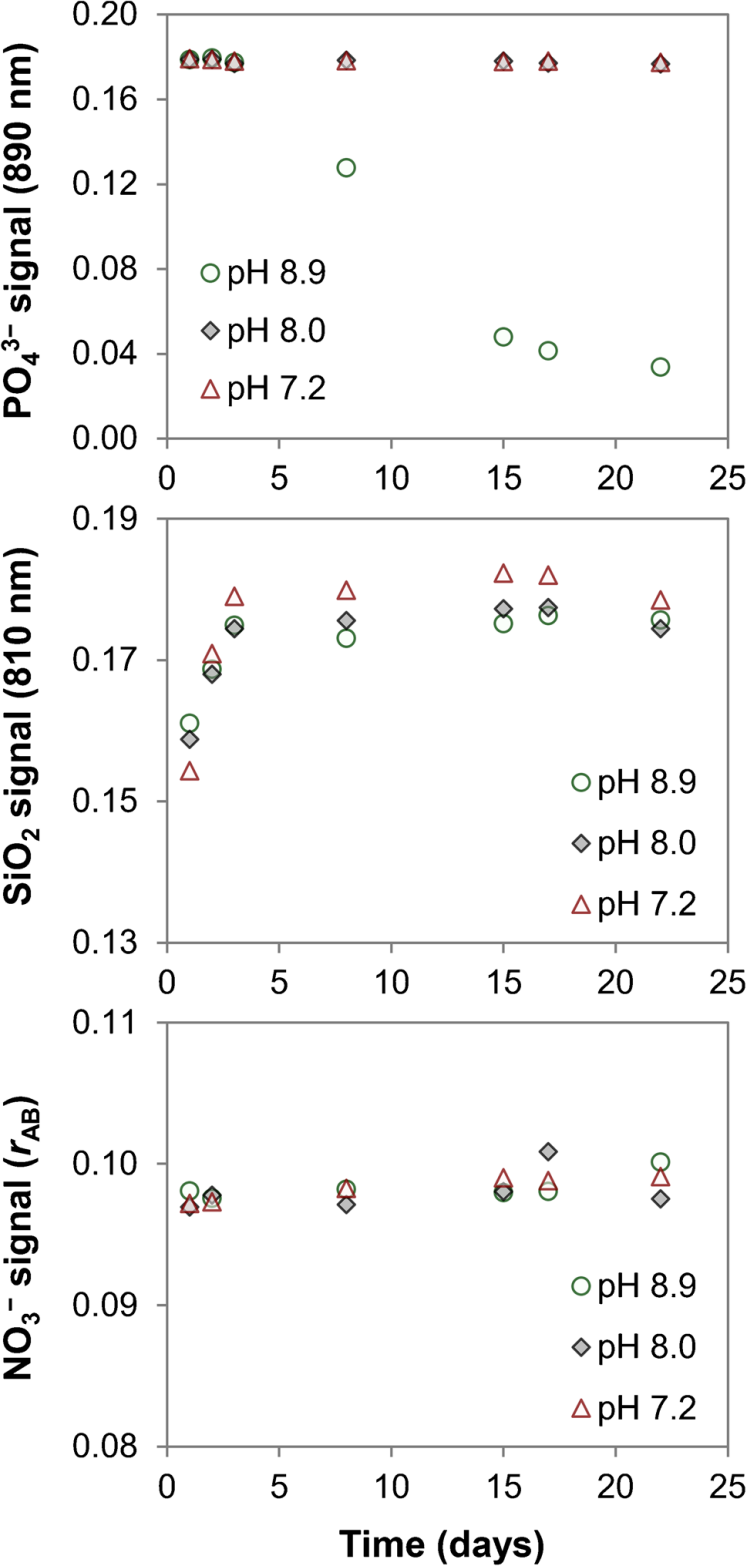
Nutrient stability in SALT-1 4.0% *w*/*w* solution: effect of pH and time. Three SALT-1 solutions were prepared in water. One was analyzed as-is (pH 8.9) and the other two were pH adjusted with 0.1 M HCl (pH 8.0 and 7.2). All samples were kept at room temperature. Top: phosphate results (photometry); middle: silicate results (photometry); bottom: nitrate results (isotope dilution GC−MS). The raw data are reported in Table S1

The effect of pH adjustment and time on the nutrient composition of 4.0% *w*/*w* SALT-1 solutions was studied. As shown in Fig. 3 (Table S1), the pH-adjusted SALT-1 solutions (pH 7.2 and 8.0) showed no systematic differences when analyzed for phosphate, silicate, and nitrate with the exception of phosphate: after the third day—in the untreated SALT-1 sample at pH 8.9—its response underwent a significant drop. This effect was likely due to a biological contamination in that sample.

As for the silicate response, the higher values obtained at pH 7.2 could be explained by the silicate blank of the 0.1 M HCl used to buffer the medium. For the methods used, pH correction was not altering nutrient response; therefore, all certification experiments were performed on the 4.0% *w*/*w* SALT-1 solution prepared in water. When the SALT-1 is used with analytical methods that are sensitive to the initial pH of the sample, 0.1 M HCl could be used to adjust the pH accordingly (Fig. 2).

**Fig. 4:**
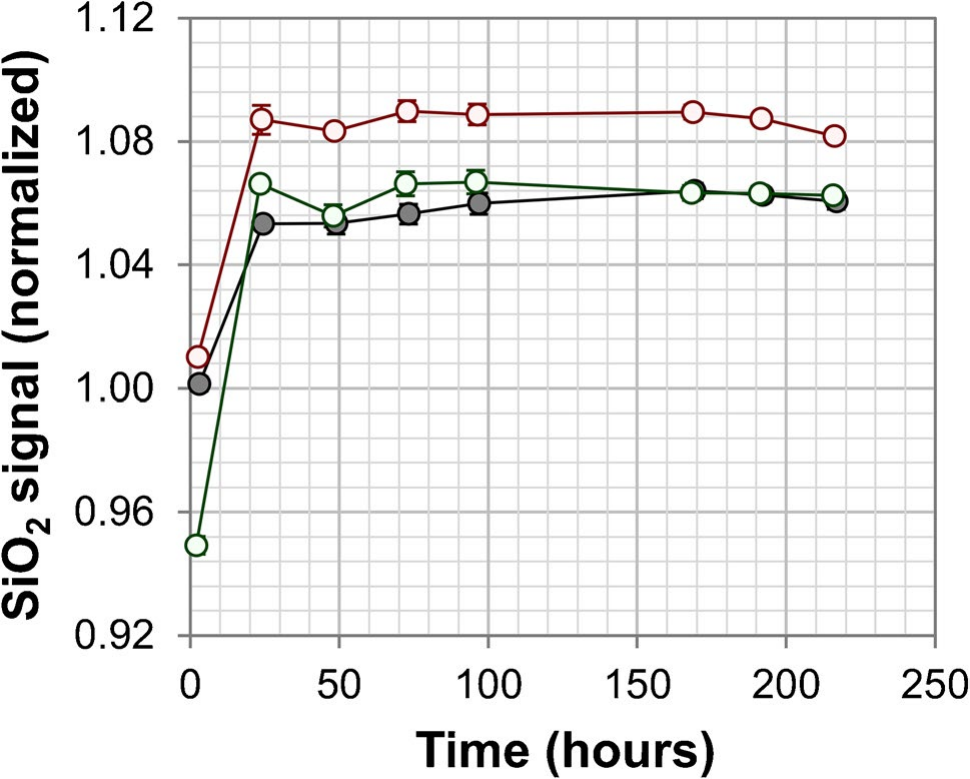
Solubility of silicate after SALT-1 reconstitution. Three 4.0% *w*/*w* SALT-1 solutions were prepared in water and kept at room temperature. Each solution was measured in triplicate at different time intervals along with a reference solution of silicate prepared in low-nutrient seawater (error bars are standard deviations, *n* = 3). The SALT-1 signal was normalized against that of the reference solution and plotted against time. The results of the three SALT-1 samples are reported in red, green, and black circles. Complete dissolution was observed after 24 h (raw data are reported in Table S2)

From Fig. 3 (Table S1), it can be noticed that the dissolution of phosphate and nitrate is instantaneous, whereas the silicate requires 24 h at room temperature (Fig. 4 and Table S2). For all quantitations described in this study, the 4.0% *w*/*w* SALT-1 solution was prepared in high-purity water and stored in PE bottles at room temperature. The solution was manually shaken to facilitate dissolution. Phosphate and nitrate were measured within 48 h after dissolution. Silicate was measured 24 h after dissolution (up to 3 days).

#### Major components: metals and anions

The artificial sea salt was chosen in order to create a material with a matrix composition similar to natural seawater. The reconstituted seawater prepared from SALT-1 (4.0% *w*/*w*) was analyzed for major components and compared to other seawater samples. Table 1 shows the results obtained by ICP−OES and ion chromatography with conductivity detection for major metals and anions normally found in seawater. For most components, the composition of the 4.0% *w*/*w* SALT-1 solution was within 20% of reference seawater samples. The only exception was boron which was present in the SALT-1 solution at a much lower level with respect to reference samples. However, boron in seawater is a trace element whose concentration can vary between 0.5 and 9.6 mg/L [49]; therefore, the difference reported in Table 1 can fit into the natural variations for the element.

**Table 1:** SALT-1 major components. Ion chromatography with conductivity detection (Cl^−^, Br^−^, SO_4_^2−^) and ICP−OES (B, Ca, K, Mg, S, Sr, Na): SALT-1 vs other seawaters

Ratio	Cl^−^	Br^−^	SO_4_^2−^	B	Ca	K	Mg	S	Sr	Na
SALT-1 vs Atlantic seawater	1.18	1.44	0.97	0.21	1.31	1.42	1.22	0.98	1.37	1.14
SALT-1 vs low-nutrient seawater	0.96	0.95	0.78	0.17	1.09	1.16	1.01	0.81	1.14	0.92

First line: ratio between the absolute signals obtained on 4.0% *w*/*w* SALT-1 solution and the one from costal Atlantic seawater sampled in Halifax on 2017-02-23. Second line: ratio between the absolute signals obtained on 4.0% *w*/*w* SALT-1 solution and low-nutrient seawater (LNSW). Before analysis, the samples were diluted 1:50 (1:100 for the analysis of sodium by ICP−OES) and injected (Paragraphs S1.6 and S1.7)

#### Trace metal screening by ICP−MS

The 4.0% *w*/*w* SALT-1 solution was also screened for common trace metals measured in seawater. The trace metal content of SALT-1 was then compared to the NRC CASS-6 and NASS-7 seawater CRMs. Table 2 reports the ratio between the trace metal content measured in the SALT-1 solution and the two CRMs. For most elements, it was noticed that SALT-1 was close in metal composition with respect to CASS-6 and NASS-7: the content of As, Cd, Co, Cr, Cu, Mo, Ni, V, and Zn in the SALT-1 solution was within one order of magnitude (higher or lower) of the metal content of either CASS-6 or NASS-7.

**Table 2:** SALT-1 trace metals by high-resolution ICP−MS: SALT-1 vs NRC CASS-6 and NASS-7 CRMs

Ratio	As	Cd	Co	Cr	Cu	Fe	Mn	Mo	Ni	Pb	U	V	Zn
SALT-1 vs CASS-6	0.31	7.73	1.07	10.7	4.12	15.9	119	0.19	9.21	328	0.017	0.35	3.61
SALT-1 vs NASS-7	0.21	10.2	5.59	5.89	11.7	74.4	372	0.18	17.2	1134	0.017	0.16	11.0

First line: ratio between the metal content measured on 4.0% *w*/*w* SALT-1 solution and NRC CASS-6 CRM. Second line: ratio between the metal content measured on 4.0% *w*/*w* SALT-1 solution and NRC NASS-7 CRM. Experimental details are provided in Paragraph S1.8

The content of Fe, Mn, and Pb in the SALT-1 solution was significantly higher than the CASS-6 and NASS-7 by an average factor of 45, 245, and 731, respectively. On the other hand, the U content of the SALT-1 solution was only 1.7% of that of CASS-6 and NASS-7. Since the mass fraction of these trace elements is well below the micrograms per gram level, the variability has a modest impact on the physical–chemical properties of the matrix.

### Homogeneity

As shown previously, the artificial sea salt used for SALT-1 preparation had similar properties as natural seawater. Since the residual nutrient content was low, the analytes were spiked into the matrix. For this purpose, 600 g of sea salt matrix with 1.5 mL of a concentrated nutrient solution were homogenized using a planetary ball mill (Fig. 1).

After processing, the SALT-1 was further homogenized and split in smaller aliquots. Sub-portioning was aimed to reduce the exposure time of the matrix to the atmospheric humidity during the final bottling process. The SALT-1 was transferred into 32 × 125-mL PE bottles and 14 of them were analyzed for bulk homogeneity. As reported in Fig. S6, the RSD on the 14 measurements was < 1% for nitrate, phosphate, and silicate. As the bulk homogeneity was satisfactory, the material was aliquoted into the final form for further investigations. The recovery of the spiked analytes was quantitative for nitrate (98%) and phosphate (101%). The recovery for silicate was 109%, likely due to the presence of endogenous silicates in the salt matrix.

The homogeneity study was then performed on the SALT-1. Sixteen units were selected from the batch of 850. The selection was performed to cover all the possible inhomogeneity sources arising from bottling (Table S3). The 16 units were analyzed in triplicate and in randomized order to eliminate the effect of any drifts. The response was normalized to the gravimetric composition of the sample blends, and the data were analyzed using a one-way ANOVA approach. The relative homogeneity components are shown in Table 3, and all calculations are reported in the ESM. Each unit of SALT-1 is intended to be used in whole, without subsampling (~ 4.1 g sample into 100 mL of solution).

**Table 3:** SALT-1 relative uncertainty components of analyte mass fraction (*k* = 1)

	*u*_short_	*u*_long_	*u*_hom_	*u*_char_	*u*_C_
Phosphate	0.00%	0.00%	0.38%	0.53%	0.65%
Silicate	0.00%	0.00%	1.2%	1.1%	1.6%
Nitrate	0.00%	0.00%	0.28%	0.95%	0.99%

*u*_short_ short-term stability; *u*_long_ long-term stability; *u*_hom_ homogeneity; *u*_C_ combined relative standard uncertainty

### Stability and redox properties

#### Nitrite oxidation

During the first exploratory experiments, nitrite was the analyte used to assess milling and homogenization process. Although the planetary ball milling (Fig. 1) was suitable to provide a highly homogenous material, significant loss of the added nitrite occurred. As shown previously, other nutrients did not degrade. It was hypothesized that during mixing at elevated temperature, nitrite could be converted to nitrate. Such an effect was studied by mass spectrometry following the conversion of ^15^N isotopically enriched nitrite. For this purpose, 90 mg of isotopically enriched Na^15^NO_2_ was dissolved in 1.5 mL of water and blended with 600 g of artificial sea salt resulting in *w*(^15^NO_2_^−^) of 100.7 mg/kg. The homogenization was performed using the planetary ball mill with the same program employed for the preparation of the SALT-1. After mixing, 59% of the added ^15^NO_2_^−^ was converted into ^15^NO_3_^−^. This result confirmed that the loss of nitrite followed an oxidative pathway. The sample was kept at room temperature and analyzed over time. After 3 days, the nitrite-to-nitrate conversion was 72%. However, no further conversion was observed after the third day when the sample was kept at room temperature (Fig. S7). To complete this series of experiments, the residual content of nitrite was measured on two aliquots of sample previously kept at 40 °C (34 days) and 105 °C (12 days). Despite the significant degradation of nitrite, 7.7 mg/kg and 0.59 mg/kg ^15^NO_2_^−^ (out of the 100.7 mg/kg originally spiked) were still found in the respective samples. The conversion of nitrite to nitrate suggests a temperature-modulated redox chemical process on the salt surface. This effect is a limit to the stability of those analytes that can undergo redox degradation.

#### Short-term stability

Short-term stability modeling extremes of transportation conditions was evaluated. Three sets of three SALT-1 units were incubated at -20 °C, 20 °C, and 40 °C, respectively. The reference SALT-1 unit was kept at 4 °C (storage condition). After 10 days, one unit of SALT-1 stored at the three different temperatures was withdrawn and placed at 4 °C along with the reference unit. This operation was then repeated after 21 and 30 days. All nine SALT-1 units along with the reference one were analyzed within repeatability conditions. Each unit was analyzed three times, and the sequence was randomized to avoid effects associated with drifts. The response of the samples was normalized to the response of the reference unit. As shown in ESM, no trends in the nutrient response were observed under typical transportation condition resulting in *u*_short_ = 0.00% as shown in Table 3.

#### Long-term stability

The SALT-1 units are stored at 4 °C enclosed in thermally sealed triple laminated bags in order to limit air exchange and potential moisture uptake. Under these conditions, issues related to the long-term stability of the material were not noticed. The material was produced in April 2018 by gravimetric addition of nutrients into the artificial sea salt. The SALT-1 was analyzed for the first time in August 2019, and the certification was performed in May 2021, following one further analysis in October 2021. As reported in Fig. S8, no significant trends in nutrient concentrations were observed; as a consequence, zero uncertainty component for the long-term stability was assigned (*u*_long_, Table 3). Monitoring of stability will continue throughout the lifecycle of the CRM.

### SALT-1 property values

#### Characterization

Fifteen units were sampled across the SALT-1 lot and used for characterization. The analyses were carried out over 3 weeks: each week, five SALT-1 units were analyzed in duplicate for phosphate, silicate, and nitrate. The replicate analyses were independent from each other: the second analysis was carried out after 24 h from the first one and a fresh set of standards was prepared for quantitation. All mathematical models for the calculation of the analytical results and the uncertainties are reported in the ESM. Briefly, nitrate results were obtained by exact matching quadruple isotope dilution following triethyloxonium derivatization [43, 50, 51], and the corresponding uncertainty was calculated by error propagation (see Paragraph S1.11 [52]). For the determination of phosphate and silicate, external calibration using standards prepared in a matrix-matched medium (low-nutrient seawater, OSIL) was employed. Some minor nonlinear trends were observed for these two analytes (Fig. S9); therefore, the standard addition calibration was not implemented [53, 54]. As reported in Fig. S10, the external calibration using matrix matching was adequate to account for rotational matrix effects. In fact, the calibration curve prepared into the 4.0% *w*/*w* SALT-1 solution was not statistically different with respect to the one obtained in low-nutrient seawater, for both phosphate and silicate. Furthermore, both the 4.0% *w*/*w* SALT-1 solution and the low-nutrient seawater medium showed similar kinetics of color development for phosphate, silicate, and nitrite (Fig. S11).

For phosphate and silicate, the calibration was obtained with a 5-level calibration plot where the middle point was exactly matched to the mass fraction of the analytes in the sample. Results were calculated using both linear (*y* = *a*_0_ + *a*_1_*x*) and quadratic (*y* = *a*_0_ + *a*_1_*x* + *a*_2_*x*^2^) model; since the relative differences between the two approaches was less than 0.3% on average (Fig. S12), the results from the more intuitive linear model were used for value assignment instead of the quadratic which offers some allowance for potential nonlinearities. For phosphate and silicate, the uncertainty on single measurement (i.e., characterization uncertainty) was estimated with both uncertainty propagation [55] and Monte Carlo approach [56]. As shown in Fig. S12, the uncertainty propagation estimate was more robust; therefore, it was used to assign the uncertainty component due to characterization (*u*_char_, Table 3). In Table 4, all approaches for the evaluation of the characterization uncertainty are compared, including the relative standard deviation from repeated measurements. Although being based on different principles, all three estimates are in a reasonable agreement.

**Table 4:** Relative standard uncertainty on nutrients mass fraction (SALT-1). Comparing different approaches for the evaluation of the characterization uncertainty component (*u*_char_). Phosphate and silicate were measured by photometry, nitrate by GC−MS

Analyte	Uncertainty propagation	Monte Carlo	RSD	*n*
Phosphate	0.53%	0.35%	0.63%	31
Silicate	1.10%	0.84%	1.6%	31
Nitrate	0.95%	N/A	0.81%	26

Uncertainty propagation: obtained applying the law of uncertainty propagation [55] (Paragraphs S1.9 and S1.11); Monte Carlo: uncertainty obtained using a Monte Carlo simulation (Paragraphs S1.10). *RSD* relative standard deviation from multiple measurements; *n* number of measurements

Two calibration standards were used for quantitation, one from NIST and one from MilliporeSigma (traceable to NIST). Within the limit of the experimental error, there were no differences between results from using the two individual sets of standards. Furthermore, within each measurement sequence, one quality control sample (MOOS-3 CRM) and one spike recovery sample were analyzed. As shown in Tables 5 and 6, quantitative recoveries were obtained.

**Table 5:** Quality control (QC) on nutrient determination by photometry (phosphate and silicate) and GC−MS (nitrate). Results obtained on the MOOS-3 CRM

Analyte	Measured, μg/g (*k* = 2)	Certified, μg/g (*k* = 2)
Phosphate (as PO_4_^3−^)	0.148 ± 0.020	0.147 ± 0.013
Silicate (as SiO_2_)	1.746 ± 0.020	1.77 ± 0.04
Nitrate (as NO_3_^−^)	1.384 ± 0.054	1.384 ± 0.015

**Table 6:** Quality control (QC) on nutrient determination by photometry (phosphate and silicate) and GC−MS (nitrate). Results obtained on the spike recovery experiment

Analyte	Recovery	RSD	*n*
Phosphate	99.4%	0.6%	7
Silicate	101.7%	1.0%	7
Nitrate	100.1%	0.5%	6

Recovery: percent of analyte recovered with respect to the gravimetric data. *RSD* relative standard deviation of the recovery; *n* number of measurements

#### Value assignment

The mass fraction of the nutrients in the SALT-1 powder is as follows: *w*(phosphate, PO_4_^3−^) = 3.744 ± 0.049 μg/g, *w*(silicate as SiO_2_) = 13.04 ± 0.42 μg/g, and *w*(nitrate, NO_3_^−^) = 28.73 ± 0.57 μg/g. These values were obtained by average of the analytical results. The corresponding uncertainty (*k* = 2) was obtained by combining the contributions due to homogeneity and characterization (zero uncertainty was assigned to short- and long-term stability). The density of the resulting 4.0% *w*/*w* SALT-1 solution was 1.02321 ± 0.00091 g/mL (*k* = 2, *n* = 16) as measured with a 25-mL pycnometer.

#### Phosphate and silicate by HPLC ICP−MS/MS

Value assignment for both phosphate and silicate was obtained using the traditional photometric methods. In order to confirm these values, an independent approach based on HPLC ICP−MS/MS was developed (Paragraph S1.5). On a total of 11 measurements by standard addition on three units of SALT-1, an average phosphate content of 3.64 ± 0.24 μg/g (RSD 6.7%, *k* = 1) and an average silicate content of 13.54 ± 0.47 μg/g (RSD 3.4%, *k* = 1) was obtained. These values are in reasonable agreement with the ones obtained by photometry as reported in the previous paragraph.

#### Reconstitution of the SALT-1

The SALT-1 was designed as a proxy for the MOOS-3 CRM [7]. Despite SALT-1 lacking nitrite, all other nutrients are close to the level found in MOOS-3. When the entire content of a SALT-1 unit was reconstituted in water to a final volume of 100 mL, the amount concentration of nutrients in the resulting solution was as reported in Table 7 [38]. Such values are within a factor of 4 with respect to the nutrient content found in the MOOS-3. Although all the testing for the certification of SALT-1 were performed at the 4.0% *w*/*w* level, the reconstitution of SALT-1 to higher (or lower) salinities would allow expanding the scope of the CRM to salinity ranges different than those of typical seawater.

**Table 7:** Certified quantity values and expanded uncertainties (*k* = 2) when the entire SALT-1 unit was reconstituted in water to a solution volume of 100 mL

Analyte	Amount concentration μmol/L	*U*_*R*_ (%)
Phosphate (as PO_4_^3−^)	1.615 ± 0.030	1.9%
Silicate (as SiO_2_)	8.89 ± 0.31	3.5%
Nitrate (as NO_3_^−^)	18.98 ± 0.45	2.4%

*U*_*R*_ is the relative combined uncertainty (%)

## Conclusion

In this study, the production strategy for a salt powder CRM (SALT-1), which is being used as a proxy for the preparation of seawater, is described. The salt matrix was spiked with a nutrient solution containing phosphate, silicate, and nitrate. A high level of homogeneity was obtained by means of a planetary ball mill. This formulation offered many advantages with respect to the traditional water-based CRMs. Notably, the preparation process was simple and economical, and could contribute to increase production/availability of CRMs for the quality control of natural waters. Furthermore, for those analytes that do not suffer oxidative degradation, the new format can offer better stability, particularity for those analytes prone to biological degradation. A further advantage of the novel CRM format is the significant decrease of its storage volume with respect to seawater CRMs: this volume reduction helps minimize the costs associated with storage and distribution.

In the future, this preparation strategy will be employed for the production of salt CRM proxies for other analytes and for matrices with lower salinity. Particularly, this novel approach could be of interest for those analytes difficult to stabilize, including many persistent organic pollutants and organometallics.

## Supplementary Information

Below is the link to the electronic supplementary material.

Supplementary file1 (PDF 7542 KB)

Supplementary file2 (XLSM 651 KB)
