# Supplementary material for: From sea salt to seawater: a novel approach for the production of water CRMs﻿
Source: Anal Bioanal Chem. 2022 May 12;414(16):4745–56. doi: 10.1007/s00216-022-04098-0 (PMC9174130; doi:10.1007/s00216-022-04098-0)
Supplement: Supplementary file 1 — Supplementary file1 (PDF 7542 KB) [file 216_2022_4098_MOESM1_ESM.pdf]

Analytical and bioanalytical chemistry

Electronic supplementary material

From sea salt to seawater: a novel approach for the production of water CRMs

Enea Pagliano, Kenny Nadeau, Ovidiu Mihai, Indumathi Pihillagawa Gedara, and Zoltán Mester

National Research Council Canada, 1200 Montreal Road, K1A0R6, Ottawa, Ontario, Canada

Email: enea.pagliano@nrc-cnrc.gc.ca

Table of Content

S1. Supporting paragraphs 3

S1.1 Photometric determination of nitrite 3

S1.2 Photometric determination of phosphate 3

S1.3 Photometric determination of silicate 4

S1.4 GC−MS determination of nitrate 5

S1.5 ICP−MS/MS for phosphate and silicate 6

S1.6 Ion chromatography conductivity for major components 6

S1.7 ICP−OES for major components 7

S1.8 ICP−MS for trace metals 7

S1.9 Determination of phosphate and silicate by photometry: uncertainty evaluation by propagation (combined standard uncertainty) 8

S1.10 Determination of phosphate and silicate by photometry: uncertainty evaluation by Monte Carlo simulation 10

S1.11 Determination of nitrate by GC−MS: uncertainty evaluation by propagation (combined standard uncertainty) 12

S2. Supporting figures 15

Figure S1. Mass of SPIN-1 material in the single units 15

Figure S2. SALT-1 moisture pick up curve ... 16
Figure S3. Time to fill each unit of SALT-1 ... 17
Figure S4. Absorbance of SALT-1 matrix as a function of pH ... 19
Figure S5. Absorbance of SALT-1 matrix under typical measurement conditions ... 21
Figure S6. SALT-1 bulk homogeneity ... 22
Figure S7. In matrix conversion of nitrite to nitrate ... 23
Figure S8. Long-term stability of nutrients in SALT-1 ... 25
Figure S9. Nonlinearity observed in nutrient analysis ... 26
Figure S10. Rotational matrix effects: SALT-1 *vs* low nutrient seawater ... 27
Figure S11. Kinetics of color development: SALT-1 *vs* low nutrient seawater ... 28
Figure S12. Quantitation of phosphate and silicate by photometry: linear *vs* quadratic model, and error propagation *vs* Monte Carlo ... 29
Figure S13. GC–MS nitrate in SALT-1 ... 30

S3. Supporting tables ... 31
Table S1. Nutrient stability in 4.0% *w*/*w* solution of SALT-1: effect of pH and time (room temperature) ... 31
Table S2. Solubility of silicate along the reconstitution of SALT-1 ... 32
Table S3. Selection of SALT-1 units for the homogeneity study ... 33

S4. References ... 34

S1. Supporting paragraphs

S1.1 Photometric determination of nitrite

The determination of nitrite was obtained by UV-vis spectroscopy using the Griess method adapted from Ref. [1, 2]. Two reagents were required for the derivatization:

a. Reagent NO2-R1. A 5.0 mL volume of concentrated (37%) hydrochloric acid was added to 30 mL of water. The solution was gently mix and allowed to cool down. 0.5 g of sulfanilamide was added and dissolved. The solution was further diluted with water to a final mass of 50.0 g. This solution was stored in a polyethylene bottle at 4 °C for one month.

b. Reagent NO2-R2. 0.1 g of *N*-(1-naphthyl)-ethylenediamine dihydrochloride was dissolved into 100 mL of water. This solution was stored in a polyethylene bottle at 4 °C for one month.

For color development, a 5 mL volume of the sample (or standard) was mixed with 0.1 mL of NO2-R1. After 5 min, 0.1 mL of NO2-R2 solution was added. The absorbance of the solution was read after 15 min (but within 2 h) at 541 nm. The derivatization was performed in plastic bottles. The absorbance of the derivatized sample was read at 541 nm (5 s integration time using 1 nm spectral bandwidth). The absorbance was measured using a quartz cuvette (1 cm).

S1.2 Photometric determination of phosphate

The determination of phosphate was obtained by UV-vis spectroscopy using the molybdenum blue method adapted from Ref. [2-6]. The reagents required for the derivatization were prepared as follow:

a. Reagent PO4-R1: sulfuric acid, [H_2SO_4] ≈ 3.8 mol/L. A 3.8 M solution of sulfuric acid was prepared by slowly adding 20 mL of concentrated sulfuric acid to 80 mL water. The solution was stored in a polyethylene bottle at room temperature for three months.

b. Reagent PO4-R2: ammonium molybdate, $(NH_4)_6Mo_7O_{24} \cdot 4H_2O$, [Mo] ≈ 0.268 mol/L. 1.42 g of ammonium molybdate was dissolved in 30 mL water. The solution was stored in a polyethylene bottle at room temperature for one month (protected from light).

c. Reagent PO4-R3: antimony potassium tartrate $C_8H_4K_2O_{12}Sb_2 \cdot xH_2O$, [Sb] ≈ 48.2 mmol/L. 0.148 g antimony potassium tartrate hydrate was dissolved in 10 mL water. The solution was stored in a polyethylene bottle at room temperature for three months.

d. Reagent PO4-MIX: [H_2SO_4] ≈ 2.8 mol/L, [Mo] ≈ 53.6 mmol/L, [Sb] ≈ 2.41 mmol/L. The combined reagent was prepared by mixing 2 mL of PO4-R2, 0.5 mL PO4-R3, and 7.5 mL PO4-R1. The solution was stored in a polyethylene bottle at room temperature and was prepared daily.

e. Reagent PO4-AA: ascorbic acid, [$C_6H_8O_6$] ≈ 0.335 mol/L. 0.59 g of ascorbic acid was dissolved in 10 mL water. The solution was stored in a polyethylene bottle at 4 °C and it was prepared daily.

f. Reagent PO4-Acid: [H_2SO_4] ≈ 2.8 mol/L, [Sb] ≈ 2.41 mmol/L. This reagent was prepared by mixing 2 mL of water, 0.5 mL PO4-R3, and 7.5 mL PO4-R1. The solution was stored in a polyethylene bottle at room temperature for three months.

For color development, a 6 mL volume of the sample (or standard) was mixed with 0.5 mL of PO4-MIX. Then, 0.2 mL of PO4-AA solution was added. The derivatization was performed in plastic bottles. After 30 min, the absorbance of the solution was read at 890 nm (5 s integration time using 1 nm spectral bandwidth). The absorbance was measured using a quartz cuvette (5 cm).

The residual absorbance of the matrix was also subtracted from the signal reading [3]. For this purpose, a 6 mL volume of the sample (or standard) was mixed with 0.5 mL of PO4-Acid. Then, 0.2 mL of PO4-AA solution was added and the absorbance of the solution was read at 890 nm.

For quantitation, five standards and a reagent blank were prepared in low nutrient seawater. Both linear and quadratic calibration curves were obtained. Uncertainty was evaluated using both Gauss [7] and Monte Carlo approach [8] (Paragraphs S1.7-S1.8 and attached excel file).

S1.3 Photometric determination of silicate

The determination of reactive silicate was obtained by UV-vis spectroscopy using the molybdenum blue method adapted from Ref. [2, 9]. The reagents required for the derivatization were prepared as follow:

a. Reagent Si-R1: sulfuric acid, [H_2SO_4] ≈ 4.7 mol/L. A 4.7 M solution of sulfuric acid was prepared by slowly adding 10 mL of concentrated sulfuric acid to 30 mL water. The solution was stored in a polyethylene bottle at room temperature for three months.

b. Reagent Si-R2: ammonium molybdate, $(NH_4)_6Mo_7O_{24} \cdot 4H_2O$, [Mo] ≈ 0.36 mol/L. 1.27 g ammonium molybdate was dissolved in 10 mL of water. After complete dissolution, 10 mL of Si-R1 was added. The solution was stored in a polyethylene bottle at room temperature for one month (protected from light).

c. Reagent Si-R3: oxalic acid, [$H_2C_2O_4 \cdot 2H_2O$] ≈ 0.79 mol/L. 1.0 g oxalic acid dehydrate was dissolved in 10 mL water. The solution was stored in a polyethylene bottle at room temperature for three months.

d. Reagent Si-AA: ascorbic acid, [$C_6H_8O_6$] ≈ 0.16 mol/L. 0.28 g ascorbic acid was dissolved in 10 mL water. The solution was stored in a polyethylene bottle at 4 °C and it was prepared daily.

e. Reagent Si-Acid: sulfuric acid, [H_2SO_4] ≈ 2.4 mol/L. 10 mL of Si-R1 were added to 10 mL of water. The solution was stored in a polyethylene bottle at room temperature for three months.

For color development, a 3 mL volume of the sample (or standard) was mixed with 0.12 mL of Si-R2. After 10 min, 0.12 mL of Si-R3 and 0.06 mL of Si-AA were added. The solution was mixed after every addition. Between addition of Si-R3 and Si-AA there was no waiting time. The derivatization was performed in plastic bottles. After 60 min, the absorbance of the solution was read at 810 nm (5 s integration time using 1 nm spectral bandwidth). The absorbance was measured using a quartz cuvette (1 cm).

The residual absorbance of the matrix was also subtracted from the signal reading [3]. For this purpose, a 3 mL volume of the sample (or standard) was mixed with 0.12 mL of Si-Acid. After 10 min, 0.12 mL of Si-R3 and 0.06 mL of Si-AA were added. The solution was mixed after every addition. Between addition of Si-R3 and Si-AA there was no waiting time. After 60 min, the absorbance of the solution was read at 810 nm.

For quantitation, five standards and a reagent blank were prepared in low nutrient seawater. Both linear and quadratic calibration curves were obtained. Uncertainty was evaluated using both Gauss [7] and Monte Carlo approach [8] (Paragraphs S1.7 and S1.8 and attached excel file).

S1.4 GC−MS determination of nitrate

The determination of nitrate was performed by headspace GC−MS using triethyloxonium tetrafluoroborate to convert NO_3^- into a volatile derivative [10-15]:

$NO_3^- + Et_3O^+ \rightarrow EtONO_2 + Et_2O$

This derivatization is a single-step aqueous derivatization. A 2.0 mL aliquot of seawater sample was transferred in a 10 mL headspace vial along with 0.2 mL of $^{15}NO_3^-$ solution of internal standard. The amount of internal standard was chosen to match the concentration of nitrate in the sample. The sample preparation was performed gravimetrically. At this point, 0.1 mL of aqueous sulfamic acid (1% *w*/*w*) was added to the mix to remove nitrite. This blend was then reacted with 50 µL of Et_3OBF_4 aqueous solution. Aqueous solutions of Et_3OBF_4 are unstable and are prepared just before derivatization. For this purpose, 1.0 g of Et_3OBF_4 (commercial reagent) was dissolved in 1.0 g of cold water (4 °C) and used within 5 min. This blend was then analyzed by headspace GC−MS. The vial was incubated at 60 °C for 2 min with agitation. A 500 µL volume of headspace was sampled (gas-tight syringe held at 70 °C) and injected in the gas chromatograph. The syringe was cleaned by flushing it with nitrogen for 5 min. The inlet liner (internal diameter of 1 mm) was held at 120 °C and the injection was performed in 7:1 split mode. Separation was obtained on a mid-polarity column (DB-624; length: 30 m; stationary phase: 6%-cyanopropyl-phenyl-94%-dimethyl polysiloxane; 0.25 mm inner diameter; 1.40 µm coating) using the following temperature program: 1.5 min (at 50 °C),

then 20 °C/min to 140 °C for a total run time of 6 min (constant flow mode: 1.0 mL He/min). The temperature of the transfer line was 220 °C. Ethyl nitrate was detected in negative chemical ionization mode (CH_4 reaction gas; CI gas flow: 40%; source and quadrupole temperature: 150 °C). The acquisition was obtained in selected ion monitoring at *m*/*z* 46 and 47 (50 ms dwell time for each ion). The GC−MS chromatogram of the ethyl nitrate derivative is shown in Fig. S13. The integration of the signal was obtained using the Agilent MassHunter software (B.06.00, Built 6.0.633.0, Agile integrator, 2012).
Nitrate quantitation was obtained using quadruple isotope dilution [13]. Calculation and uncertainty evaluation can be found in Paragraph S1.9 and in the attached excel file.

S1.5 ICP−MS/MS for phosphate and silicate

An Agilent 1200 series HPLC (Agilent Technologies, Mississauga, Ontario) with an ion exclusion column Dionex IonPac ICE-AS1 (4 x 250 mm) was used to separate phosphate and silicate from the major ions in sea water samples. A PEEK tubing (0.13 mm ID x 1.59 mm OD) was used to connect the column of the HPLC to the ICP−MS/MS introduction system. The sample uptake was 25 µL and the eluent used in the experiment was HCl 0.01% and the flow rate was 0.25 mL/min.
All measurement were made with an Agilent 8800 (Agilent Technologies, Mississauga, Ontario) operating in hydrogen mode to measured mass 28 (Si) and 31 (P) on an 8 min acquisition window. A Ga 20 ng/g internal standard solution was simultaneously introduced to monitor the stability of the signal. Hydrochloric acid used for the eluent was purified in-house and the deionized water was from a Thermo Scientific Gen-Pure UV xCAD plus system (18.2 MΩ cm at 25 °C). The standards SRM 3150 silicon solution (lot number: 130912) was from the National Institute of Standards and Technology (NIST, Gaithersburg, MD) and the Supelco phosphate standard 38364 (lot number: BCCD8182) was from Sigma Aldrich (Oakville, ON, Canada). These primary standards were used for the preparation of the standard addition calibration curves.
Three different samples of SALT-1 were prepared by dissolving approximatively 4.0 g of sample into 100 g of water. Freshly prepared samples were allowed to rest for 24 hours at room temperature. There aliquots from each samples were prepared and spiked respectively to achieve the standard addition calibration curves (one un-spiked sample and two spiked samples to double and triple the signal of the sample). The blank used was DIW and was also spiked to match the signal observed in the SALT-1.

S1.6 Ion chromatography conductivity for major components

Chloride, bromide, and sulfate were detected by ion chromatography with conductivity detection. A Thermo Scientific ion chromatography system was used. A single pump ICS-5000^+ SP-5 was interfaced with an AS-AP autosampler (p/n 074925). Detection was performed by suppressed conductivity. A Thermo Scientific conductivity detector (p/n 061830) was used on-line with a Dionex self-regenerating suppressor (AERS 500, 4 mm, p/n 082540). Ion separation was obtained with the Dionex IonPack AS23 column (4x250 mm) equipped with the Dionex AG23 guard column (4x50 mm). A 25 µL sample volume was injected and elution was performed in 25 min in isocratic mode (4.5 mM Na_2CO_3, 0.8 mM $NaHCO_3$) at 1.0 mL/min flow rate and 30 °C. A 25 mA current was applied to the

suppressor and conductivity detection was attained at 35 °C (20 Hz sample collection rate). For sample preparation, the seawater was simply diluted 1:50 before injection.

S1.7 ICP–OES for major components

Preliminary screening was performed for sodium, boron, magnesium, calcium, potassium, sulfur and strontium using Agilent 5110 SVDV ICP–OES. Synchronous Vertical Dual View (SVDV) was used for the measurement of the target analytes. For each individual measurement SVDV combines both axial and radial light from the plasma, working together with a high speed VistaChip II CCD detector. The argon plasma was maintained at 1.20 kW RF power, with 12.0 L/min argon plasma flow and 1.0 L/min argon auxiliary flow. The salt solutions were nebulized into the plasma using the ICP–OES built in peristaltic pump and an argon nebulizer flow of 0.7L/min. Analysis was performed at the following elemental wavelengths: sodium (589.592nm), boron (249.772nm), magnesium (279.800nm), calcium (317.933nm), potassium (766.491nm), sulfur (180.669nm, 181.972nm), strontium (407.771nm). For sample preparation, the seawater was simply diluted 1:50 before injection; for sodium analysis the dilution was 1:100.

S1.8 ICP–MS for trace metals

A seaFAST from Elemental Scientific (ESI, Omaha, NE) was used as an automated preconcentration system to separate analytes of interest in undiluted seawater samples. The column used in the seaFAST was the CF-N-0200 and it was coupled to the Thermo ELEMENT XR (Bremen, Germany) sector field ICP–MS. About 6.0 mL of sample was injected by the seaFAST autosampler. The analytes of interest were concentrated on the column and then eluted to the ICP–MS. The analytes measured were Cd, Co, Cu, Fe, Mn, Ni, Pb, V and Zn. Some analytes like Mo and U did not need pre-concentration and were measured after simple dilution. For Cr measurement, samples were reduced using hydroxylamine hydrochloride solution, then separated and pre-concentrated offline using a column of silica immobilized diphenylcarbazone. Samples were prepared by diluting approximatively 4.0 g of SALT-1 in 100 g of a solution of nitric acid at pH of 1.6. A three points standard addition calibration was used for As, Cd, Co, Cu, Fe, Mn, Pb, Ni, V and Zn. Isotope dilution calibration was used for Cr, Mo and U. Deionized water (DIW) was obtain from a Nanopure ion exchange reverse osmosis system (Barnstead/Thermolyne, Bosta, MA). Nitric acid was purified in-house using a sub-boiling distillation system. The standard stock solutions were prepared from high-purity metals with purity determined by the NRC GD–MS facility. A SRM 3164 uranium stock solution from National Institute of Standards and Technology (NIST, Gaithersburg, MD) was used. Seawater CRMs NASS-7 and CASS-6 from National Research Council (NRC, Ottawa, Canada) were used as quality control.

S1.9 Determination of phosphate and silicate by photometry: uncertainty evaluation by propagation (combined standard uncertainty)

Both phosphate and silicate were measured by photometry following quantitation against an external calibration curve (5-points) obtained in matrix-matched low nutrient seawater. For the estimation of the combined standard uncertainty on the final results we followed the JCGM 100 guide [7]. For a general model equation $y = f(x_1, x_2, \cdots, x_n)$ the combined variance on y is given by this equation:

$$u_c^2(y) = \sum_{i=1}^{N}\sum_{j=1}^{N}\frac{\partial f}{\partial x_i}\cdot\frac{\partial f}{\partial x_j}\cdot u(x_i, x_j) = \sum_{i=1}^{N}\left(\frac{\partial f}{\partial x_i}\right)^2\cdot u^2\,(x_i) + 2\cdot\sum_{i=1}^{N-1}\sum_{j=i+1}^{N}\frac{\partial f}{\partial x_i}\cdot\frac{\partial f}{\partial x_j}\cdot u(x_i, x_j)$$

In the case of external calibration with linear model ($y = a_0 + a_1 \cdot x$), the only correlated variables are the intercept and slope of the calibration curve. Their correlation coefficient is estimated as follow [16]:

$$r(a_0, a_1) = \frac{u(a_0, a_1)}{u(a_0)\cdot u(a_1)} = \frac{-\bar{x}\cdot u^2(a_1)}{u(a_0)\cdot u(a_1)} = -\bar{x}\cdot\frac{u(a_1)}{u(a_0)}$$

The combined standard uncertainty on the result was calculate using an Excel VBA function, as reported in the attached Excel file. VBA code of the function:

```
Function GaussLinearUNC(a0_ As Variant, _
                        ua0_ As Variant, _
                        a1_ As Variant, _
                        ua1_ As Variant, _
                        r_ As Variant, _
                        y_ As Variant, _
                        uy_ As Variant, _
                        w_ As Variant, _
                        uw_ As Variant) As Double

'Calculation uncertainty Gauss method on the OLS with linear model combined
'with the uncertainty of the primary standard
'Equation: y = a0 + a1x
'Solution: x = (y - a0)/a1
'Primary standard: w with uw uncertainty

Dim a0 As Double      'intercept
Dim ua0 As Double     'uncertainty on intercept
Dim a1 As Double      'slope
Dim ua1 As Double     'uncertainty on slope
Dim r As Double       'correlation coefficient intercept slope
Dim y As Double       'signal of the sample
Dim uy As Double      'uncertainty on the signal of the sample
Dim w As Double       'mass fraction of the primary standard
Dim uw As Double      'uncertainty on the mass fraction of the primary standard
```

```
a0 = a0_
ua0 = ua0_
a1 = a1_
ua1 = ua1_
r = r_
y = y_
uy = uy_
w = w_
uw = uw_

Dim x As Double        'mass fraction of the sample
Dim ux As Double       'uncertainty on the mass fraction of the sample
Dim GaussU As Double   'uncertainty of the x without the contribution of the primary
standard
Dim RSD As Double      'relative uncertainty on x (total)

x = (y - a0) / a1

GaussU = Sqr((1 / a1) ^ 2 * (uy ^ 2 + ua0 ^ 2) + ((a0 - y) / a1 ^ 2) ^ 2 * ua1 ^ 2 _
         - 2 * (1 / a1) * ((a0 - y) / a1 ^ 2) * ua0 * ua1 * r)

RSD = Sqr((GaussU / x) ^ 2 + (uw / w) ^ 2)

GaussLinearUNC = x * RSD

End Function
```

S1.10 Determination of phosphate and silicate by photometry: uncertainty evaluation by Monte Carlo simulation

Both phosphate and silicate were measured by photometry following quantitation against an external calibration curve (5-points) obtained in matrix-matched low nutrient seawater. The uncertainty on the final result was also estimated using a Monte Carlo method. Uncertainty on the *x*-axis (mass fraction) was evaluated by uncertainty propagation on the gravimetrically prepared standards. Uncertainty on the *y*-axis (normalized signals) was estimated from the ANOVA study for homogeneity: the within-group variance was used as an estimate for the uncertainty on *y*-axis.

The Monte Carlo uncertainty on the result was calculated using an Excel VBA function, as reported in the attached Excel file. VBA code of the function:

```
Function ExCAL(y_ As Variant, uy_ As Variant, x_ As Variant, ux_ As Variant, it_ As
Integer, Sample_ As Double, uSample_ As Double) As Variant

Dim Line_x As Integer
Dim Column_x As Integer
Dim Line_y As Integer
Dim Column_y As Integer
Dim Line_ux As Integer
Dim Column_ux As Integer
Dim Line_uy As Integer
Dim Column_uy As Integer
Dim l As Integer
Dim k As Integer
Dim it As Integer

Dim x As Variant
Dim ux As Variant
Dim y As Variant
Dim uy As Variant
Dim Sample As Variant
Dim uSample As Variant

'Input data transfer to local variables
x = x_
ux = ux_
y = y_
uy = uy_
Sample = Sample_
uSample = uSample_
it = it_

'Size of the variables
Line_x = UBound(x, 1) - LBound(x, 1) + 1
Column_x = UBound(x, 2) - LBound(x, 2) + 1

Line_y = UBound(y, 1) - LBound(y, 1) + 1
Column_y = UBound(y, 2) - LBound(y, 2) + 1
```

```
Line_ux = UBound(ux, 1) - LBound(ux, 1) + 1
Column_ux = UBound(ux, 2) - LBound(ux, 2) + 1

Line_uy = UBound(uy, 1) - LBound(uy, 1) + 1
Column_uy = UBound(uy, 2) - LBound(uy, 2) + 1

'The Monte Carlo simulation
If (Line_x <> Line_y) Or (Line_x <> Line_ux) Or (Line_x <> Line_uy) Or (Column_x <>
1) Or (Column_y <> 1) Or (Column_ux <> 1) Or (Column_uy <> 1) Then
    ExCAL = "N/A"
Else
    Randomize (10)
    Dim sR() As Double
    Dim LocX() As Double
    Dim LocY() As Double
    Dim LocSample As Double
    ReDim sR(1 To it)
    ReDim LocX(1 To Line_x)
    ReDim LocY(1 To Line_y)
    For l = 1 To it
        For k = 1 To Line_x
            LocX(k) = WorksheetFunction.NormInv(Rnd, x(k, 1), ux(k, 1))
            LocY(k) = WorksheetFunction.NormInv(Rnd, y(k, 1), uy(k, 1))
        Next k
        LocSample = WorksheetFunction.NormInv(Rnd, Sample, uSample)
        sR(l) = (LocSample - WorksheetFunction.Intercept(LocY, LocX)) /
WorksheetFunction.Slope(LocY, LocX)
    Next l
    ExCAL = Array(WorksheetFunction.Average(sR), WorksheetFunction.StDev(sR))
End If

End Function
```

S1.11 Determination of nitrate by GC–MS: uncertainty evaluation by propagation (combined standard uncertainty)

Quantitation of nitrate was obtained by exact-matching quadruple isotope dilution as reported previously [13, 14]. Value assignment and uncertainty evaluation (combined standard uncertainty by propagation [7]) were obtained by applying the following Excel VBA function:

```
Function unID4MS(CalMatrix As Object, SampleVector As Object) As Variant

    Dim Result, Variance, uncertainty As Variant
    'Uncertainty calculated as first-order Taylor expansion

    'A*B-1 Vector
    C5 = CalMatrix(1)
    D5 = CalMatrix(2)
    E5 = CalMatrix(3)
    F5 = CalMatrix(4)
    G5 = CalMatrix(5)
    H5 = CalMatrix(6)
    I5 = CalMatrix(7)
    J5 = CalMatrix(8)

    'A*B-2 Vector
    C6 = CalMatrix(9)
    D6 = CalMatrix(10)
    E6 = CalMatrix(11)
    F6 = CalMatrix(12)
    G6 = CalMatrix(13)
    H6 = CalMatrix(14)
    I6 = CalMatrix(15)
    J6 = CalMatrix(16)

    'A*B-3 Vector
    C7 = CalMatrix(17)
    D7 = CalMatrix(18)
    E7 = CalMatrix(19)
    F7 = CalMatrix(20)
    G7 = CalMatrix(21)
    H7 = CalMatrix(22)
    I7 = CalMatrix(23)
    J7 = CalMatrix(24)

    'AB Vector
    C10 = SampleVector(1)
    D10 = SampleVector(2)
    E10 = SampleVector(3)
    F10 = SampleVector(4)
    G10 = SampleVector(5)
    H10 = SampleVector(6)
```

```
    'Variance components of the combined uncertainty, uc(wA) : First-order Taylor
expansion

    'rab
    L10 = (E10 ^ 2 * H10 ^ 2 * (G5 - G6) ^ 2 * (G5 - G7) ^ 2 * (G6 - G7) ^ 2 * (C5 *
E6 * I5 - C6 * E5 * I6) ^ 2 * (C5 * E7 * I5 - C7 * E5 * I7) ^ 2 * (C6 * E7 * I6 - C7
* E6 * I7) ^ 2) / (C10 ^ 2 * (C5 * E6 * E7 * (G10 - G5) * (G6 - G7) * I5 + E5 * (-(C6
* E7 * (G10 - G6) * (G5 - G7) * I6) + C7 * E6 * (G5 - G6) * (G10 - G7) * I7)) ^ 4)
    'ra*b-1
    M10 = (E10 ^ 2 * (G10 - G6) ^ 2 * (G10 - G7) ^ 2 * (G6 - G7) ^ 2 * H5 ^ 2 * (C5 *
E6 * I5 - C6 * E5 * I6) ^ 2 * (C5 * E7 * I5 - C7 * E5 * I7) ^ 2 * (C6 * E7 * I6 - C7
* E6 * I7) ^ 2) / (C10 ^ 2 * (C5 * E6 * E7 * (G10 - G5) * (G6 - G7) * I5 + E5 * (-(C6
* E7 * (G10 - G6) * (G5 - G7) * I6) + C7 * E6 * (G5 - G6) * (G10 - G7) * I7)) ^ 4)
    'ra*b-2
    N10 = (E10 ^ 2 * (G10 - G5) ^ 2 * (G10 - G7) ^ 2 * (G5 - G7) ^ 2 * H6 ^ 2 * (C5 *
E6 * I5 - C6 * E5 * I6) ^ 2 * (C5 * E7 * I5 - C7 * E5 * I7) ^ 2 * (C6 * E7 * I6 - C7
* E6 * I7) ^ 2) / (C10 ^ 2 * (C5 * E6 * E7 * (G10 - G5) * (G6 - G7) * I5 + E5 * (-(C6
* E7 * (G10 - G6) * (G5 - G7) * I6) + C7 * E6 * (G5 - G6) * (G10 - G7) * I7)) ^ 4)
    'ra*b-3
    O10 = (E10 ^ 2 * (G10 - G5) ^ 2 * (G10 - G6) ^ 2 * (G5 - G6) ^ 2 * H7 ^ 2 * (C5 *
E6 * I5 - C6 * E5 * I6) ^ 2 * (C5 * E7 * I5 - C7 * E5 * I7) ^ 2 * (C6 * E7 * I6 - C7
* E6 * I7) ^ 2) / (C10 ^ 2 * (C5 * E6 * E7 * (G10 - G5) * (G6 - G7) * I5 + E5 * (-(C6
* E7 * (G10 - G6) * (G5 - G7) * I6) + C7 * E6 * (G5 - G6) * (G10 - G7) * I7)) ^ 4)
    'wA*-1
    P10 = (E10 ^ 2 * C5 ^ 2 * E5 ^ 2 * (G10 - G6) ^ 2 * (G5 - G6) ^ 2 * (G10 - G7) ^
2 * (G5 - G7) ^ 2 * (C6 * E7 * I6 - C7 * E6 * I7) ^ 4 * J5 ^ 2) / (C10 ^ 2 * (C5 * E6
* E7 * (G10 - G5) * (G6 - G7) * I5 + E5 * (-(C6 * E7 * (G10 - G6) * (G5 - G7) * I6) +
C7 * E6 * (G5 - G6) * (G10 - G7) * I7)) ^ 4)
    'wA*-2
    Q10 = (E10 ^ 2 * C6 ^ 2 * E6 ^ 2 * (G10 - G5) ^ 2 * (G5 - G6) ^ 2 * (G10 - G7) ^
2 * (G6 - G7) ^ 2 * (C5 * E7 * I5 - C7 * E5 * I7) ^ 4 * J6 ^ 2) / (C10 ^ 2 * (C5 * E6
* E7 * (G10 - G5) * (G6 - G7) * I5 + E5 * (-(C6 * E7 * (G10 - G6) * (G5 - G7) * I6) +
C7 * E6 * (G5 - G6) * (G10 - G7) * I7)) ^ 4)
    'wA*-3
    R10 = (E10 ^ 2 * C7 ^ 2 * E7 ^ 2 * (G10 - G5) ^ 2 * (G10 - G6) ^ 2 * (G5 - G7) ^
2 * (G6 - G7) ^ 2 * (C5 * E6 * I5 - C6 * E5 * I6) ^ 4 * J7 ^ 2) / (C10 ^ 2 * (C5 * E6
* E7 * (G10 - G5) * (G6 - G7) * I5 + E5 * (-(C6 * E7 * (G10 - G6) * (G5 - G7) * I6) +
C7 * E6 * (G5 - G6) * (G10 - G7) * I7)) ^ 4)
    'mA(AB)
    S10 = (D10 ^ 2 * E10 ^ 2 * (C6 * C7 * E5 * (G10 - G5) * (G6 - G7) * I6 * I7 + C5
* I5 * (C6 * E7 * (G5 - G6) * (G10 - G7) * I6 - C7 * E6 * (G10 - G6) * (G5 - G7) *
I7)) ^ 2) / (C10 ^ 4 * (C5 * E6 * E7 * (G10 - G5) * (G6 - G7) * I5 + E5 * (-(C6 * E7
* (G10 - G6) * (G5 - G7) * I6) + C7 * E6 * (G5 - G6) * (G10 - G7) * I7)) ^ 2)
    'mB(AB)
    T10 = (F10 ^ 2 * (C6 * C7 * E5 * (G10 - G5) * (G6 - G7) * I6 * I7 + C5 * I5 * (C6
* E7 * (G5 - G6) * (G10 - G7) * I6 - C7 * E6 * (G10 - G6) * (G5 - G7) * I7)) ^ 2) /
(C10 ^ 2 * (C5 * E6 * E7 * (G10 - G5) * (G6 - G7) * I5 + E5 * (-(C6 * E7 * (G10 - G6)
* (G5 - G7) * I6) + C7 * E6 * (G5 - G6) * (G10 - G7) * I7)) ^ 2)
    'mA*(A*B)-1
    U10 = (E10 ^ 2 * D5 ^ 2 * E5 ^ 2 * (G10 - G6) ^ 2 * (G5 - G6) ^ 2 * (G10 - G7) ^
2 * (G5 - G7) ^ 2 * I5 ^ 2 * (C6 * E7 * I6 - C7 * E6 * I7) ^ 4) / (C10 ^ 2 * (C5 * E6
* E7 * (G10 - G5) * (G6 - G7) * I5 + E5 * (-(C6 * E7 * (G10 - G6) * (G5 - G7) * I6) +
C7 * E6 * (G5 - G6) * (G10 - G7) * I7)) ^ 4)
    'mA*(A*B)-2
```

```
    V10 = (E10 ^ 2 * D6 ^ 2 * E6 ^ 2 * (G10 - G5) ^ 2 * (G5 - G6) ^ 2 * (G10 - G7) ^
2 * (G6 - G7) ^ 2 * I6 ^ 2 * (C5 * E7 * I5 - C7 * E5 * I7) ^ 4) / (C10 ^ 2 * (C5 * E6
* E7 * (G10 - G5) * (G6 - G7) * I5 + E5 * (-(C6 * E7 * (G10 - G6) * (G5 - G7) * I6) +
C7 * E6 * (G5 - G6) * (G10 - G7) * I7)) ^ 4)
    'mA*(A*B)-3
    W10 = (E10 ^ 2 * D7 ^ 2 * E7 ^ 2 * (G10 - G5) ^ 2 * (G10 - G6) ^ 2 * (G5 - G7) ^
2 * (G6 - G7) ^ 2 * (C5 * E6 * I5 - C6 * E5 * I6) ^ 4 * I7 ^ 2) / (C10 ^ 2 * (C5 * E6
* E7 * (G10 - G5) * (G6 - G7) * I5 + E5 * (-(C6 * E7 * (G10 - G6) * (G5 - G7) * I6) +
C7 * E6 * (G5 - G6) * (G10 - G7) * I7)) ^ 4)
    'mB(A*B)-1
    X10 = (E10 ^ 2 * C5 ^ 2 * F5 ^ 2 * (G10 - G6) ^ 2 * (G5 - G6) ^ 2 * (G10 - G7) ^
2 * (G5 - G7) ^ 2 * I5 ^ 2 * (C6 * E7 * I6 - C7 * E6 * I7) ^ 4) / (C10 ^ 2 * (C5 * E6
* E7 * (G10 - G5) * (G6 - G7) * I5 + E5 * (-(C6 * E7 * (G10 - G6) * (G5 - G7) * I6) +
C7 * E6 * (G5 - G6) * (G10 - G7) * I7)) ^ 4)
    'mB(A*B)-2
    Y10 = (E10 ^ 2 * C6 ^ 2 * F6 ^ 2 * (G10 - G5) ^ 2 * (G5 - G6) ^ 2 * (G10 - G7) ^
2 * (G6 - G7) ^ 2 * I6 ^ 2 * (C5 * E7 * I5 - C7 * E5 * I7) ^ 4) / (C10 ^ 2 * (C5 * E6
* E7 * (G10 - G5) * (G6 - G7) * I5 + E5 * (-(C6 * E7 * (G10 - G6) * (G5 - G7) * I6) +
C7 * E6 * (G5 - G6) * (G10 - G7) * I7)) ^ 4)
    'mB(A*B)-3
    Z10 = (E10 ^ 2 * C7 ^ 2 * F7 ^ 2 * (G10 - G5) ^ 2 * (G10 - G6) ^ 2 * (G5 - G7) ^
2 * (G6 - G7) ^ 2 * (C5 * E6 * I5 - C6 * E5 * I6) ^ 4 * I7 ^ 2) / (C10 ^ 2 * (C5 * E6
* E7 * (G10 - G5) * (G6 - G7) * I5 + E5 * (-(C6 * E7 * (G10 - G6) * (G5 - G7) * I6) +
C7 * E6 * (G5 - G6) * (G10 - G7) * I7)) ^ 4)


    Result = (E10 * (-C6 * C7 * E5 * (G10 - G5) * (G6 - G7) * I6 * I7 + C5 * I5 * (-
C6 * E7 * (G5 - G6) * (G10 - G7) * I6 + C7 * E6 * (G10 - G6) * (G5 - G7) * I7))) /
(C10 * (C5 * E6 * E7 * (G10 - G5) * (G6 - G7) * I5 + E5 * (-C6 * E7 * (G10 - G6) * (G5
- G7) * I6 + C7 * E6 * (G5 - G6) * (G10 - G7) * I7)))
    Variance = L10 + M10 + N10 + O10 + P10 + Q10 + R10 + S10 + T10 + U10 + V10 + W10
+ X10 + Y10 + Z10
    uncertainty = Sqr(Variance)

    unID4MS = Array(Result, uncertainty, L10 / Variance, M10 / Variance, N10 / Variance,
O10 / Variance, P10 / Variance, Q10 / Variance, R10 / Variance, S10 / Variance, T10 /
Variance, U10 / Variance, V10 / Variance, W10 / Variance, X10 / Variance, Y10 /
Variance, Z10 / Variance)

End Function
```

S2. Supporting figures

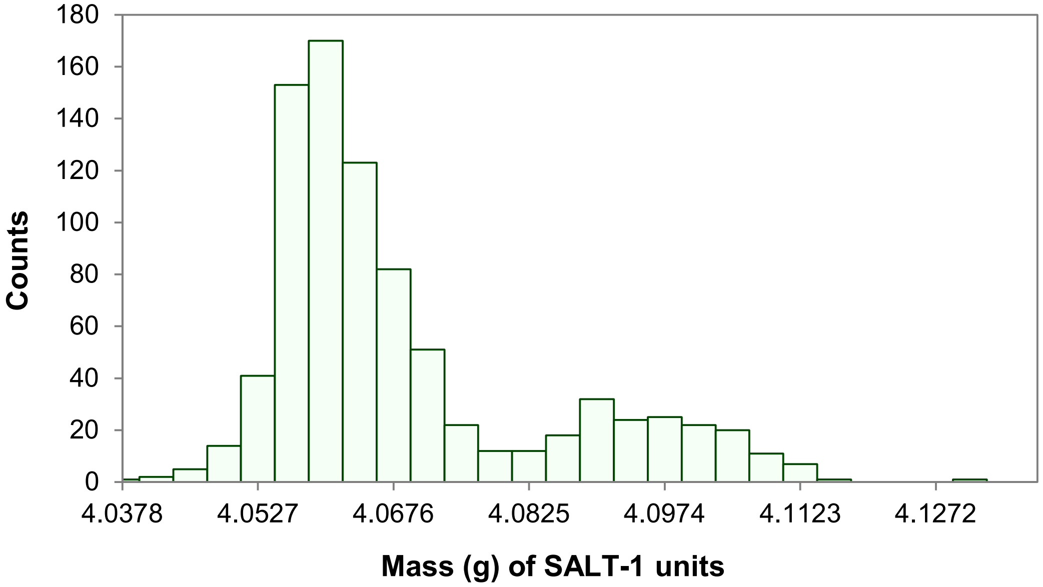

Figure S1. Mass of SPIN-1 material in the single units

Min = 4.0378 g; max = 4.1309 g; mean = 4.0668 g; standard deviation = 0.0156 g

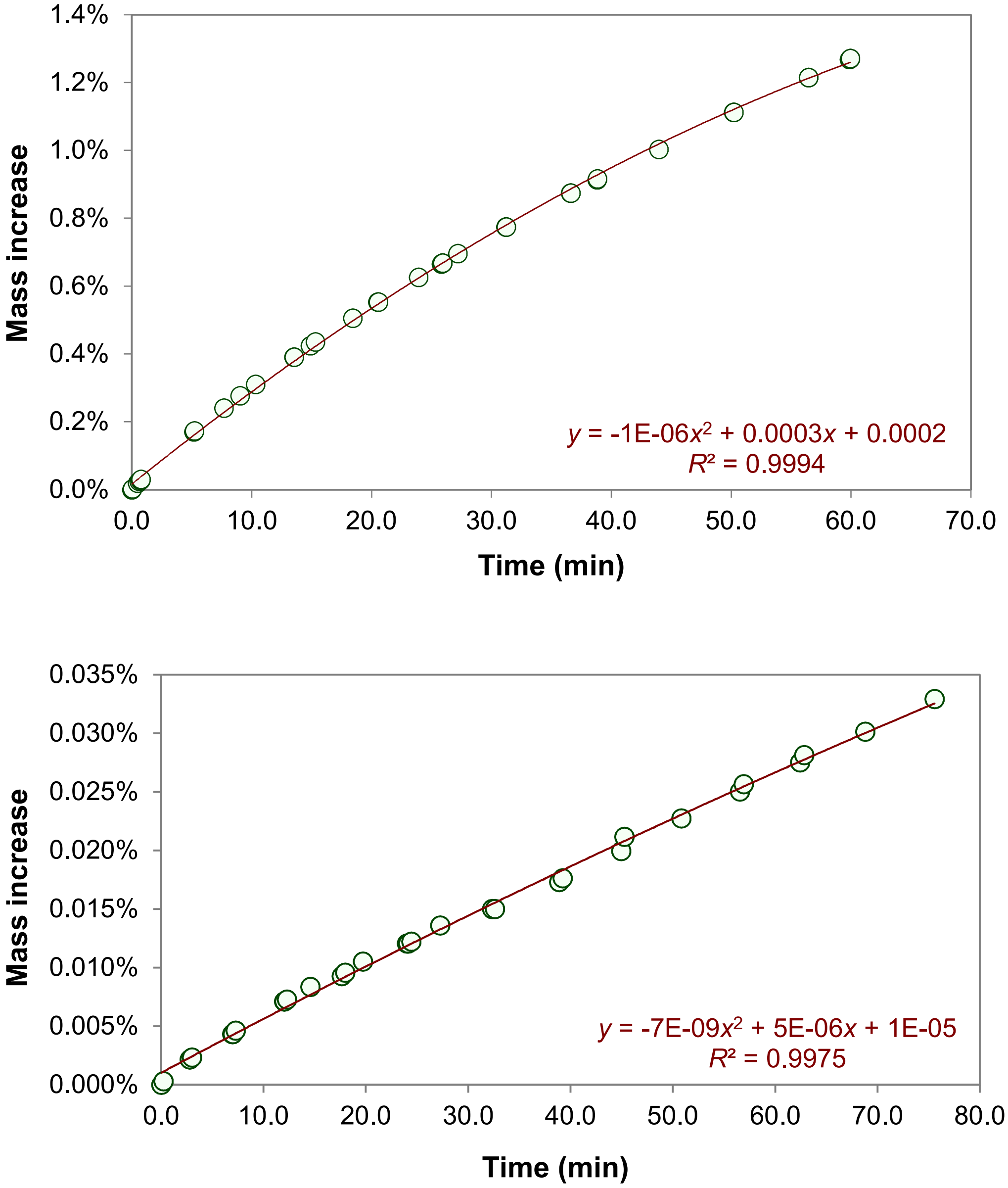

Figure S2. SALT-1 moisture pick up curve

Top: 3.5 g of SALT-1 matrix was spread on a Petri dish. Bottom: 65 g of SALT-1 matrix was transferred in a 125 mL open bottle. In both cases the mass increase (due to moisture adsorption) was monitored against time. Laboratory conditions: t = 20 °C, RH 40-60%.

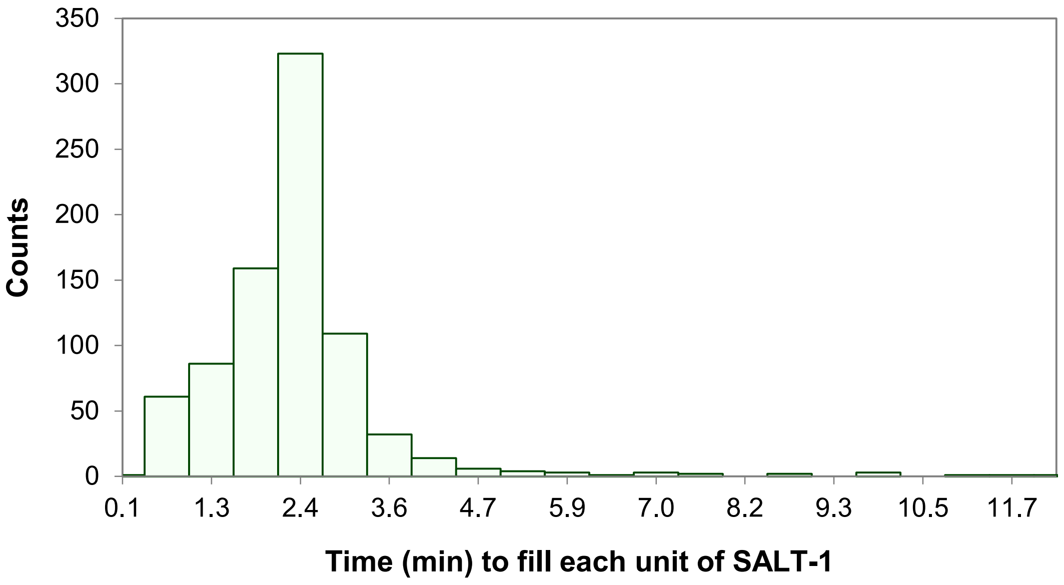

Figure S3. Time to fill each unit of SALT-1

The average time to fill a salt unit was ~2.1 min. Min = 0.12 min; max = 11.6 min; standard deviation = 1.2 min. Laboratory conditions: t = 20 °C, RH 40-60%.

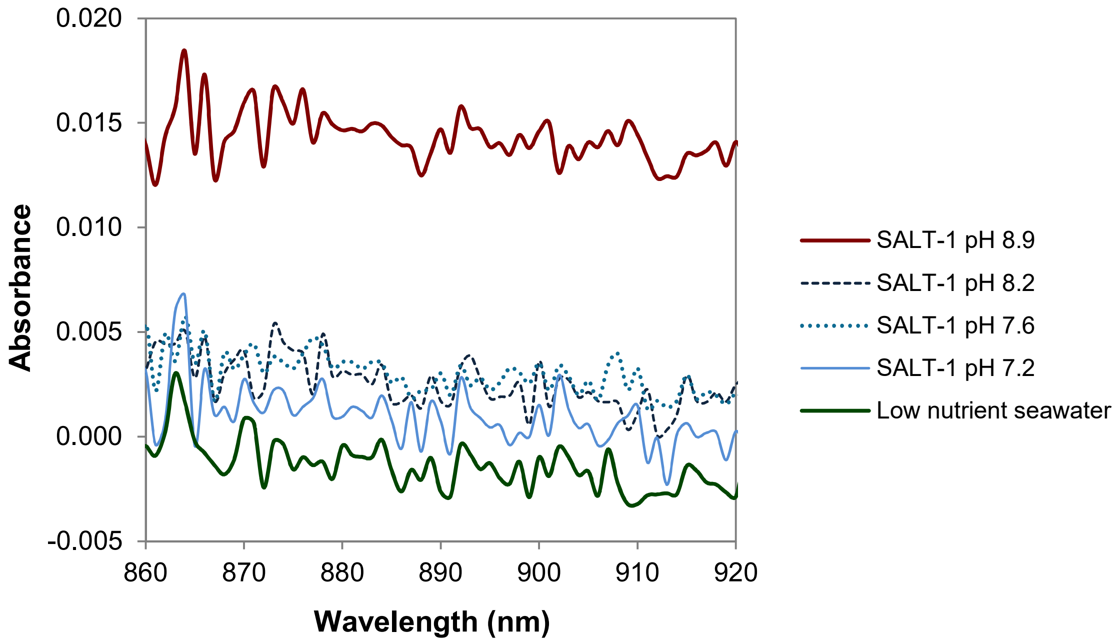
0.020
0.015
0.010
0.005
0.000
-0.005
Absorbance
860
870
880
890
900
910
920
Wavelength (nm)
SALT-1 pH 8.9
SALT-1 pH 8.2
SALT-1 pH 7.6
SALT-1 pH 7.2
Low nutrient seawater

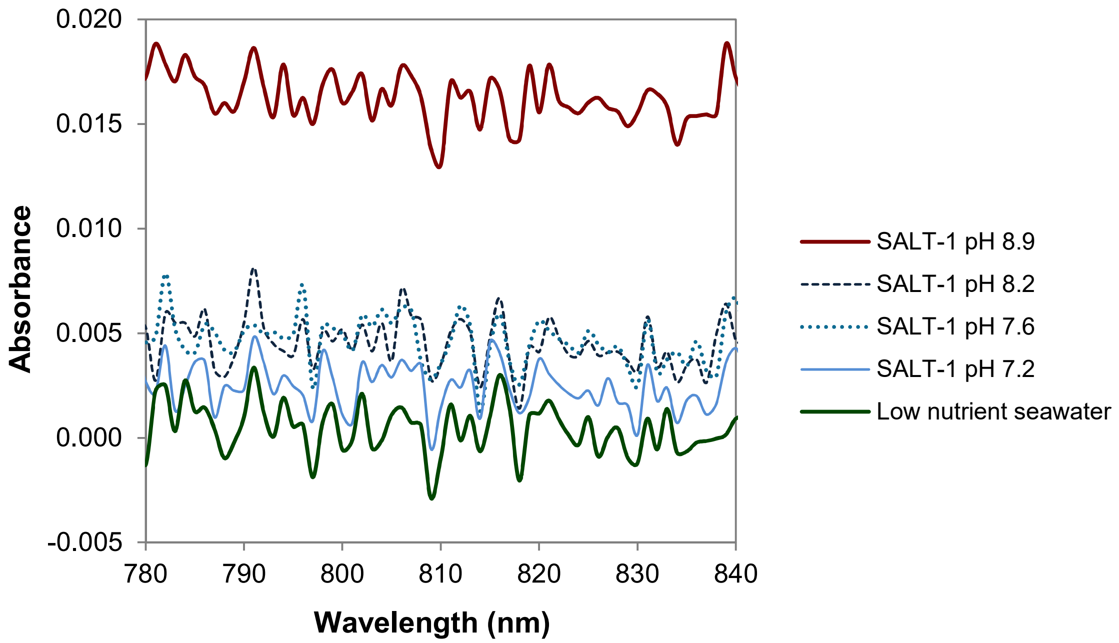
0.020
0.015
0.010
0.005
0.000
-0.005
Absorbance
780
790
800
810
820
830
840
Wavelength (nm)
SALT-1 pH 8.9
SALT-1 pH 8.2
SALT-1 pH 7.6
SALT-1 pH 7.2
Low nutrient seawater

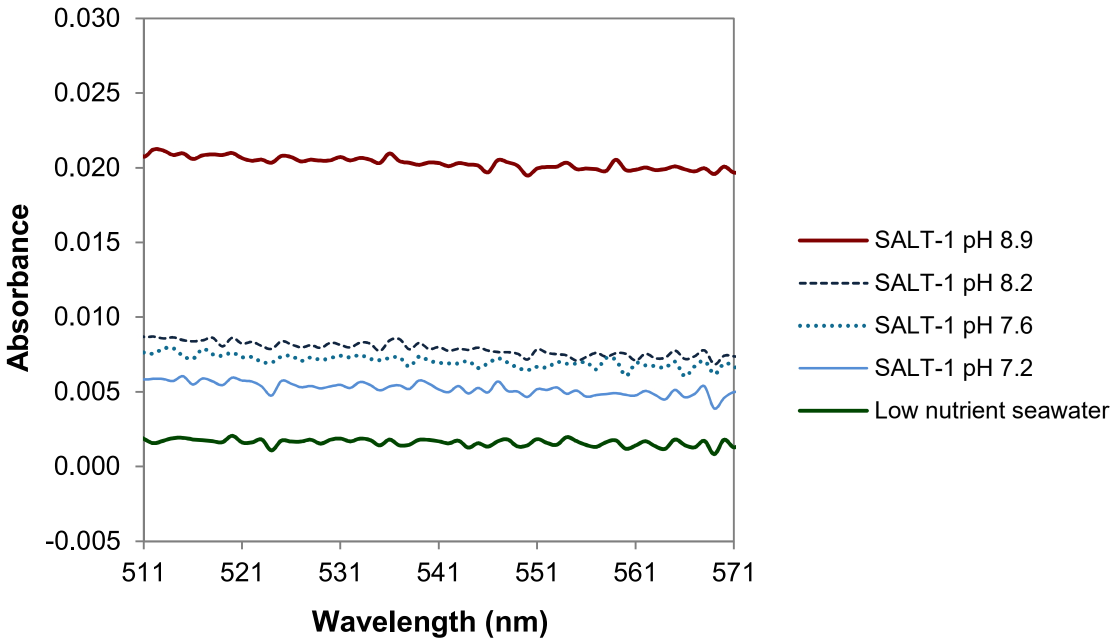

Figure S4. Absorbance of SALT-1 matrix as a function of pH

When the SALT-1 is solubilized in water (4.0% *w*/*w*) the pH of the solution is ~8.9 with a minor opalescence which cause a positive reading of absorbance. The absorbance reported in the figures was measure using a 5 cm quartz cuvette. When the pH of the SALT-1 was corrected to lower values (8.2, 7.6, 7.2), the opalescence disappeared and the residual absorbance of the solution was comparable to that of the low nutrient seawater from OSIL.

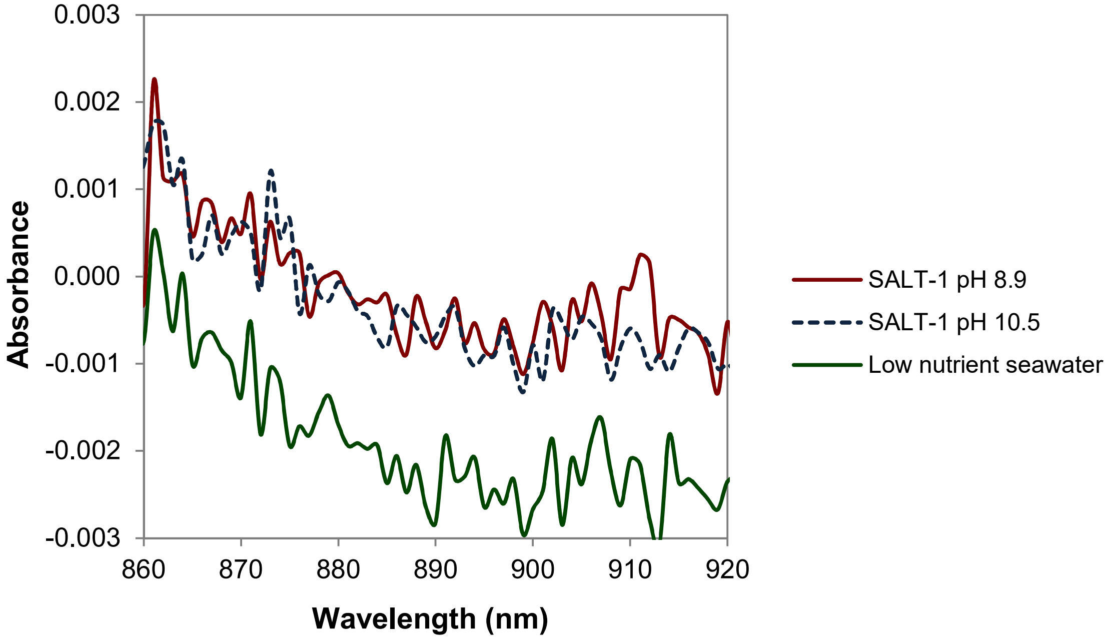
0.003
0.002
0.001
0.000
-0.001
-0.002
-0.003
Absorbance
860
870
880
890
900
910
920
Wavelength (nm)
SALT-1 pH 8.9
SALT-1 pH 10.5
Low nutrient seawater

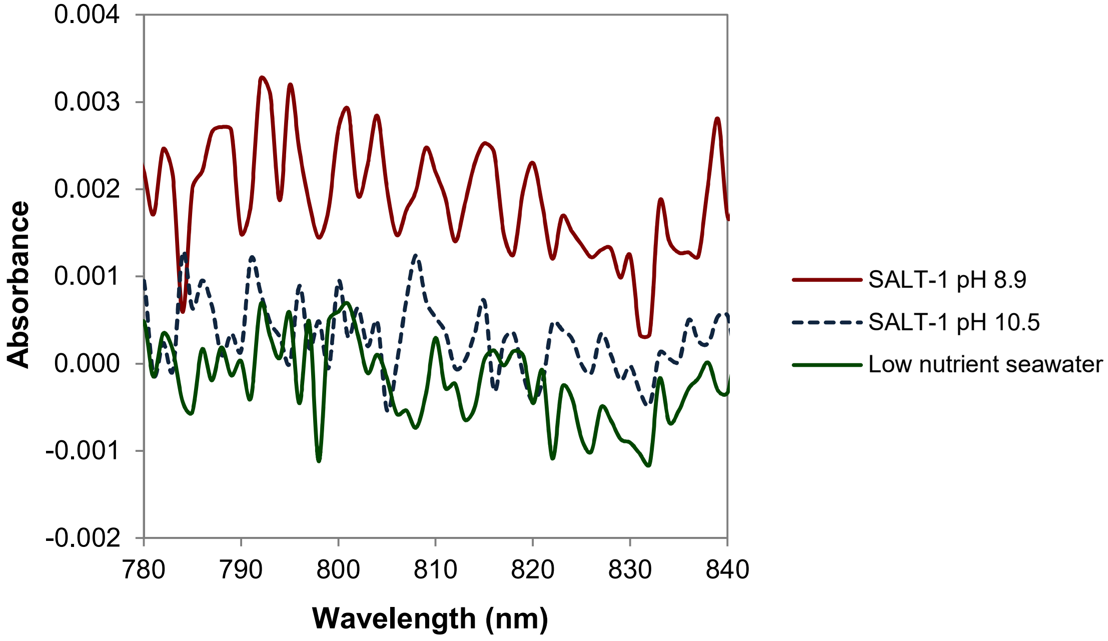
0.004
0.003
0.002
0.001
0.000
-0.001
-0.002
Absorbance
780
790
800
810
820
830
840
Wavelength (nm)
SALT-1 pH 8.9
SALT-1 pH 10.5
Low nutrient seawater

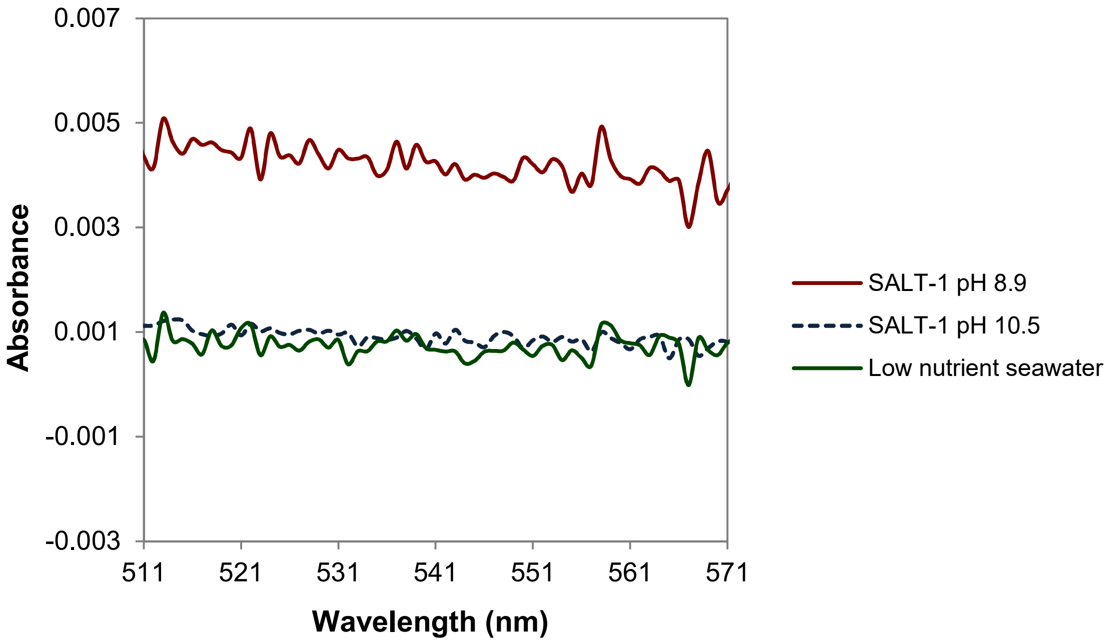

Figure S5. Absorbance of SALT-1 matrix under typical measurement conditions

The determination of phosphate, silicate, and nitrate by photometry requires derivatization under acidic conditions [2]. In this experiment, a SALT-1 sample was solubilized in water at the 4.0% *w*/*w* level (pH 8.9). Another unit of SALT-1 was baked at 105 °C for 21 days. The reconstituted solution of this sample showed a pH of 10.5 and it was quite turbid. The third (reference) sample was the low nutrient seawater from OSIL.
Each of these three samples was acidified under typical conditions for determination of phosphate, silicate, and nitrite (Paragraphs S 1.1 to S 1.3 "residual absorbance of the matrix"). The absorbance reported in the figures was measured using a 5 cm quartz cuvette. In the top graph the three samples were prepared under conditions for phosphate measurement; the middle one for silicate; the bottom one for nitrite.

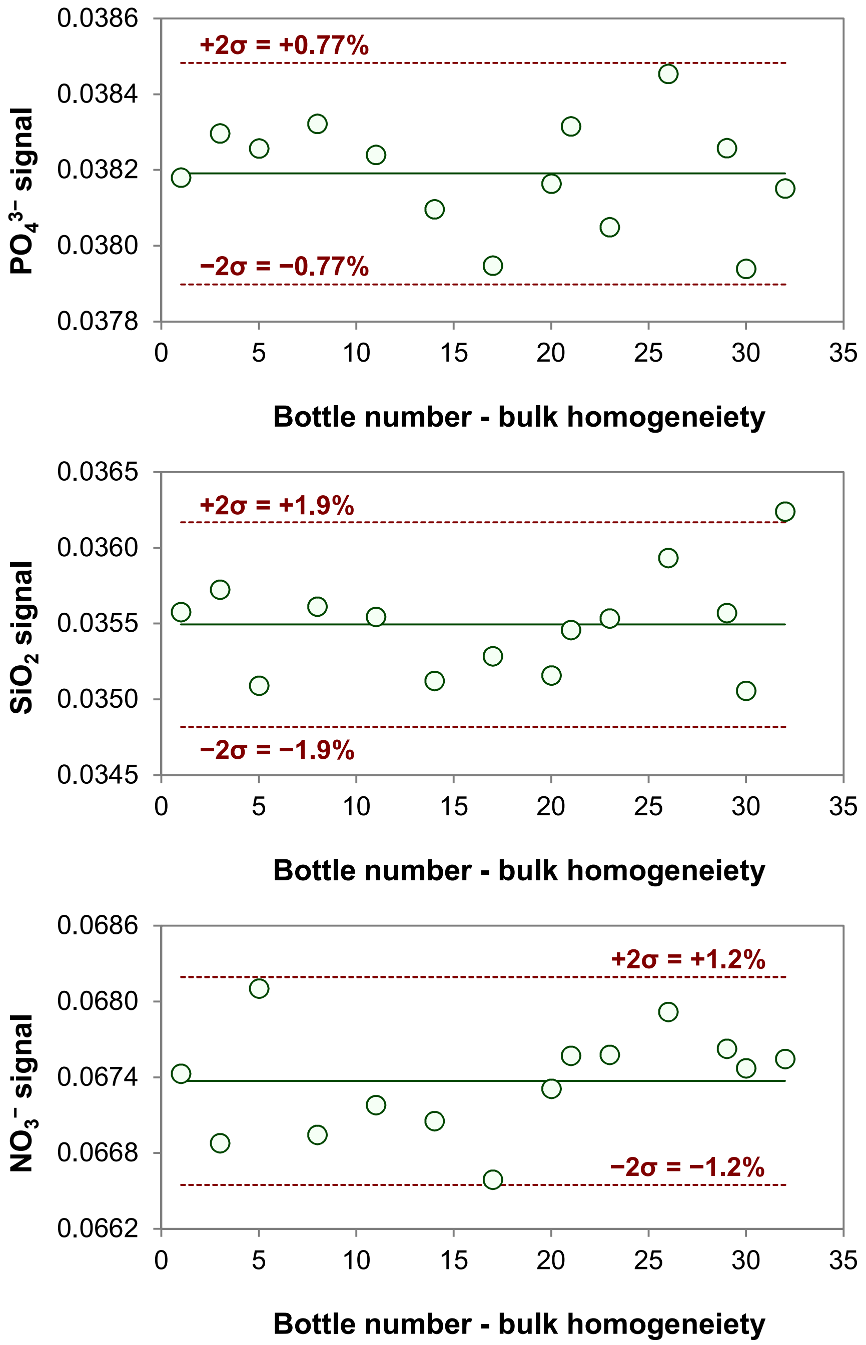

Figure S6. SALT-1 bulk homogeneity

The material was portioned in 32 x 100 mL bottles and 14 samples were analyzed once in a repeatability experiment. A RSD < 1% (*n* = 14) was obtained for all analytes.

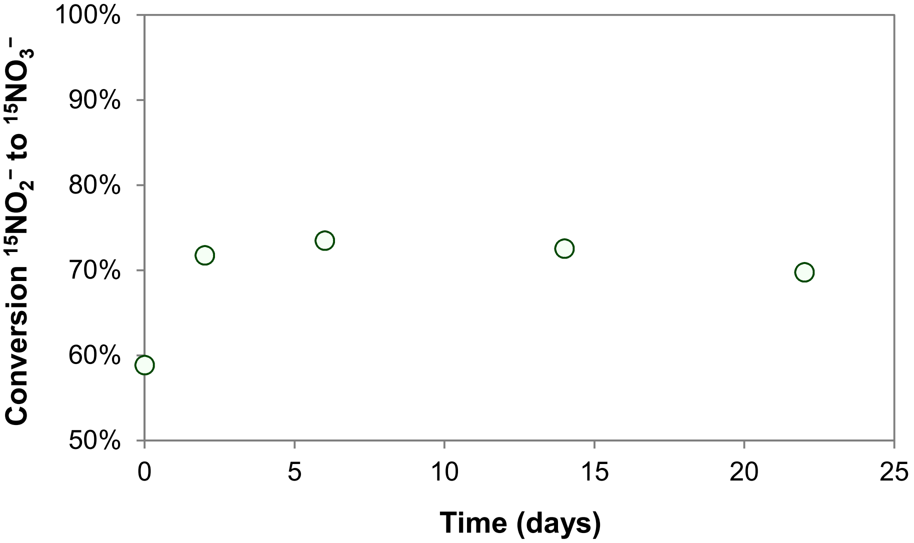

Figure S7. In matrix conversion of nitrite to nitrate

90 mg of isotopically enriched $Na^{15}NO_2$ was dissolved in 1.5 mL of water and mixed (planetary ball mill) with 600 g of aquarium salt. After mixing, 59% of the added $^{15}NO_2^-$ was converted into $^{15}NO_3^-$ (determination by GC–MS). The sample was kept at room temperature and analyzed at different times. After 2 days, the nitrite to nitrate conversion was 72%. No more conversion was observed thereafter.

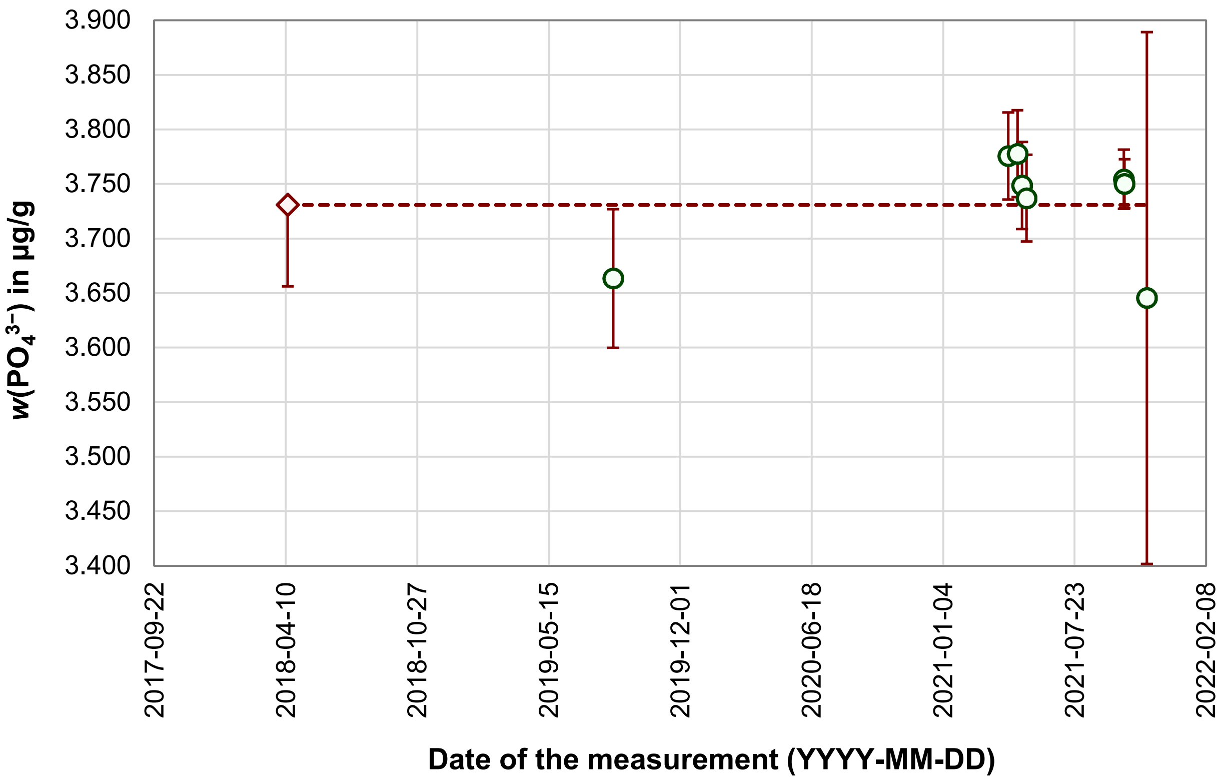

3.900
3.850
3.800
3.750
3.700
3.650
3.600
3.550
3.500
3.450
3.400
w(PO4 3−) in µg/g
2017-09-22
2018-04-10
2018-10-27
2019-05-15
2019-12-01
2020-06-18
2021-01-04
2021-07-23
2022-02-08
Date of the measurement (YYYY-MM-DD)

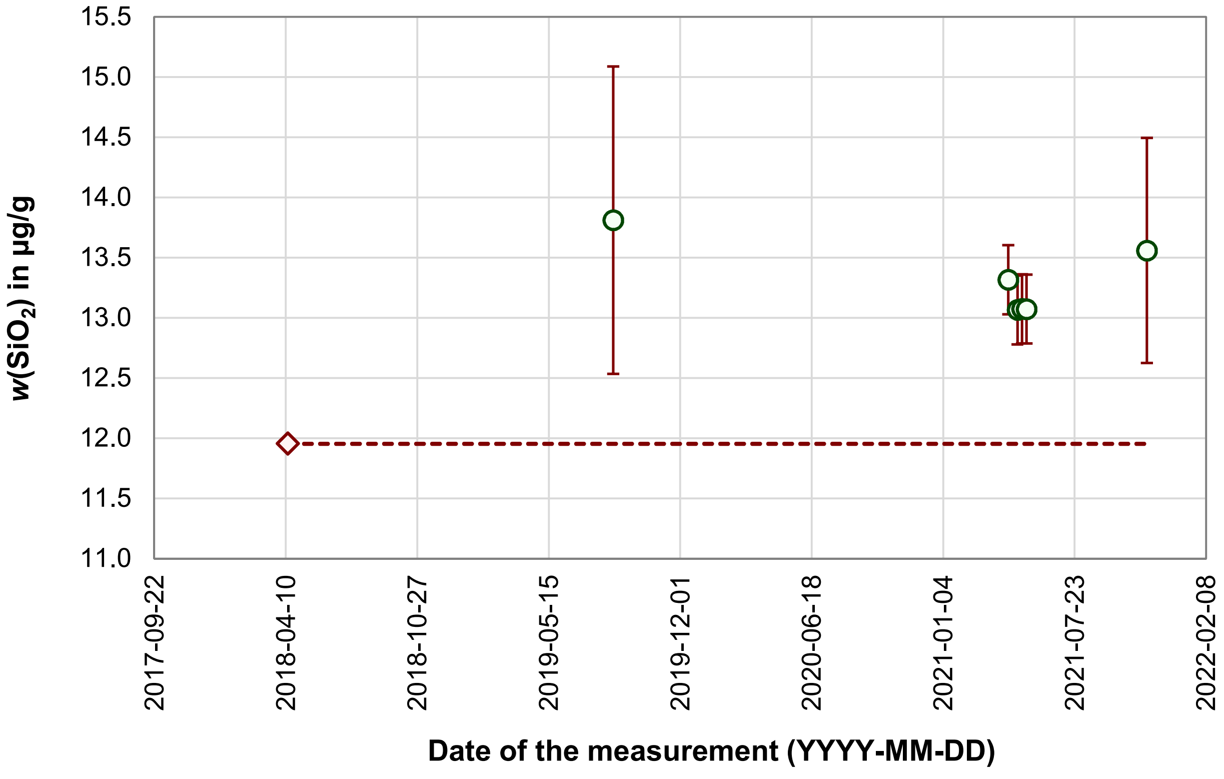

15.5
15.0
14.5
14.0
13.5
13.0
12.5
12.0
11.5
11.0
w(SiO2) in µg/g
2017-09-22
2018-04-10
2018-10-27
2019-05-15
2019-12-01
2020-06-18
2021-01-04
2021-07-23
2022-02-08
Date of the measurement (YYYY-MM-DD)

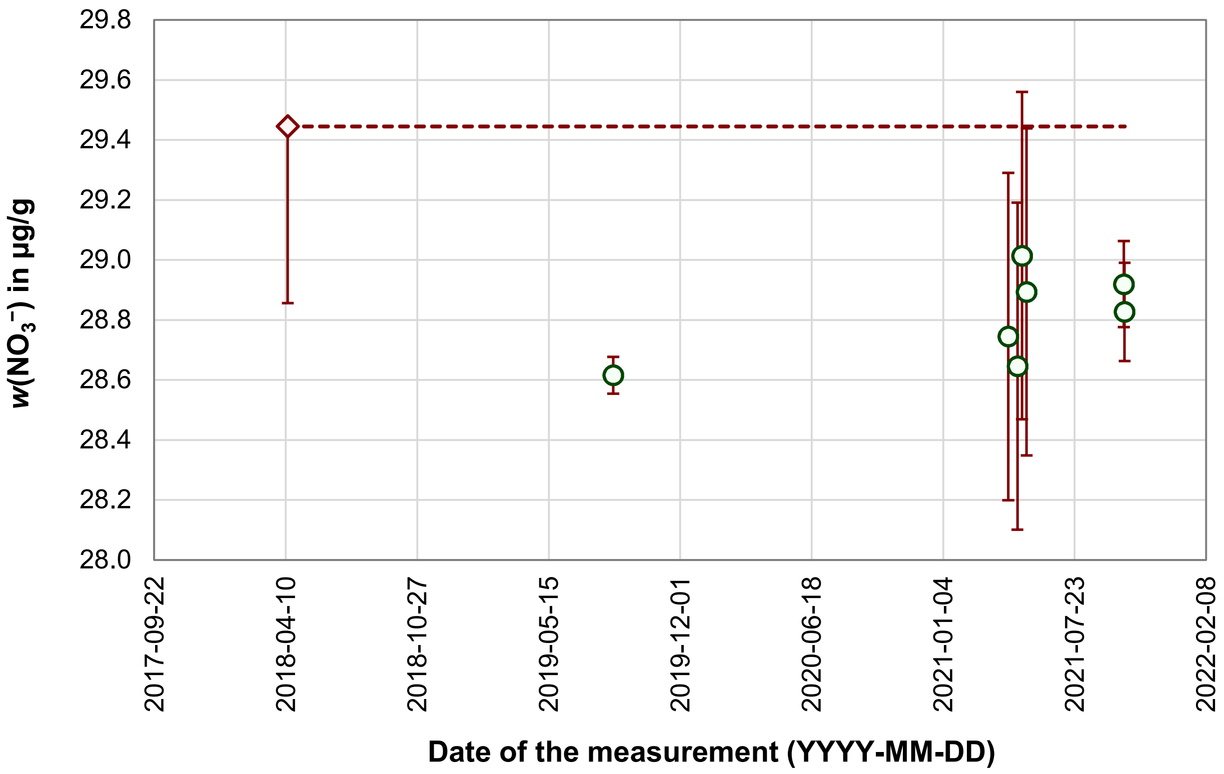

Figure S8. Long-term stability of nutrients in SALT-1

Top: phosphate; Middle: silicate; bottom: nitrate. The material was prepared on April 2018 and spiked with a certain amount of nutrients (red diamond, and red dotted line). The nutrient content was then measured in 2019 and 2021. Error bars: combined expanded uncertainty (k = 2).

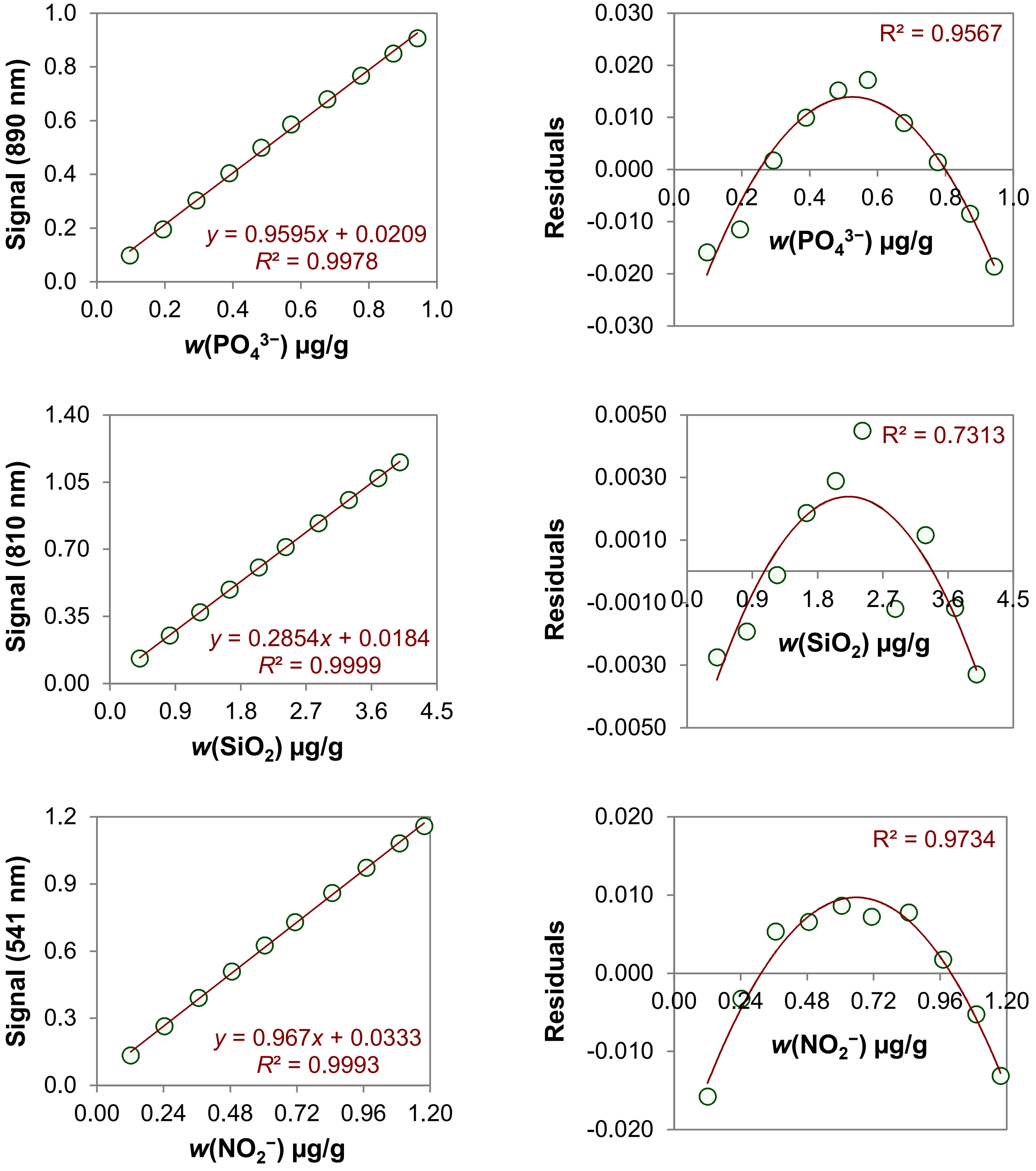

Figure S9. Nonlinearity observed in nutrient analysis

The photometric response of phosphate, silicate and nitrite was studied in the [0.1, 1] absorbance interval. 10 standard solutions were prepared in low nutrient seawater and the response was measured as described in Paragraphs S1.1 to S 1.3. Although the calibration plots are "visually" linear with R^2 > 0.997, the residual graphs shows a nonlinear response.

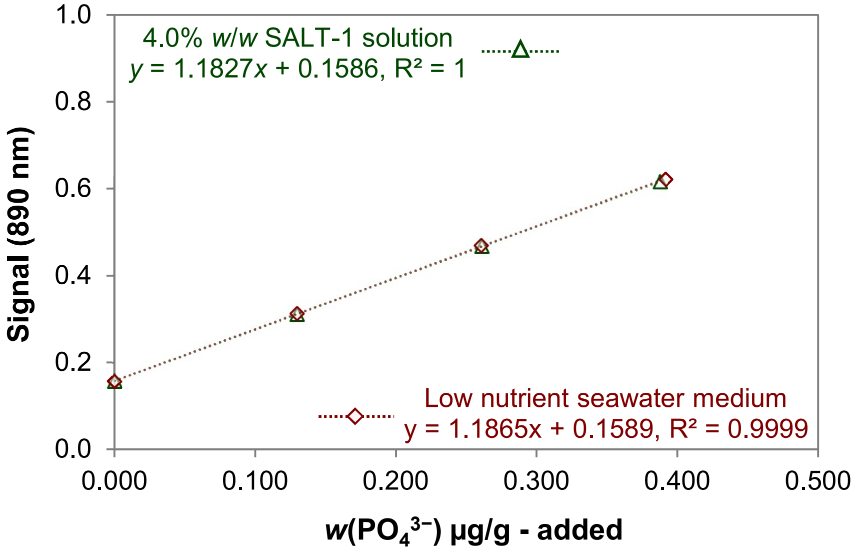

Phosphate analysis
LINEST result

SALT-1 medium

slope	*intercept*
1.1827	0.1586
0.0041	0.0010

LNSW medium

slope	*intercept*
1.1865	0.1589
0.0081	0.0020

Ratio between slopes:
0.997 ± 0.015 *k* = 2

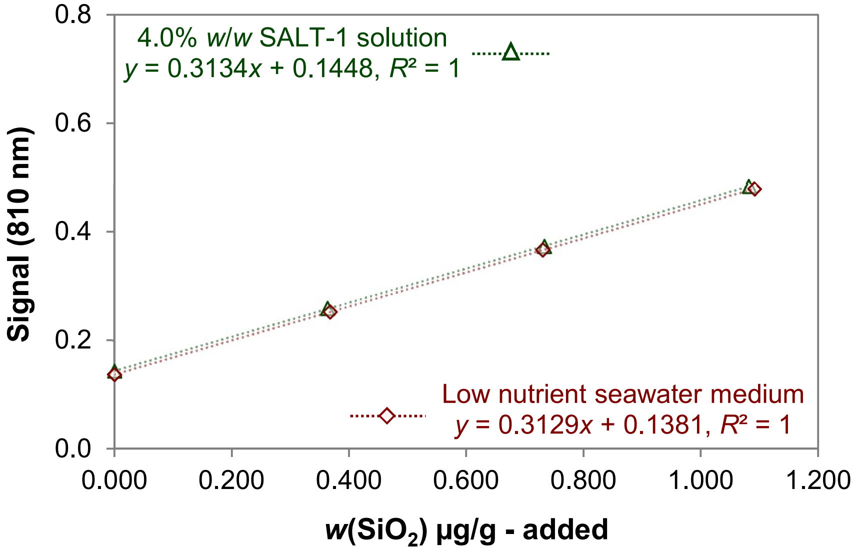

Silicate analysis
LINEST result

SALT-1 medium

slope	*intercept*
0.3134	0.14484
0.0010	0.00070

LNSW medium

slope	*intercept*
0.31294	0.13811
0.00083	0.00056

Ratio between slopes:
1.0014 ± 0.0085 *k* = 2

Figure S10. Rotational matrix effects: SALT-1 *vs* low nutrient seawater

One SALT-1 sample was reconstituted in water at the 4.0% *w*/*w* level. One nutrient solution was prepared in low nutrient seawater to closely match SALT-1 concentration for phosphate and silicate. Both materials were analyzed *as-is* and after increasing levels of phosphate and silicate. The ratio between the slopes of the calibration curve obtained in the SALT-1 medium was not significantly different than the one obtained in low nutrient seawater for both analytes. No relevant (rotational) matrix effects were observed. Therefore, the matrix-matching external calibration was used for quantitation.

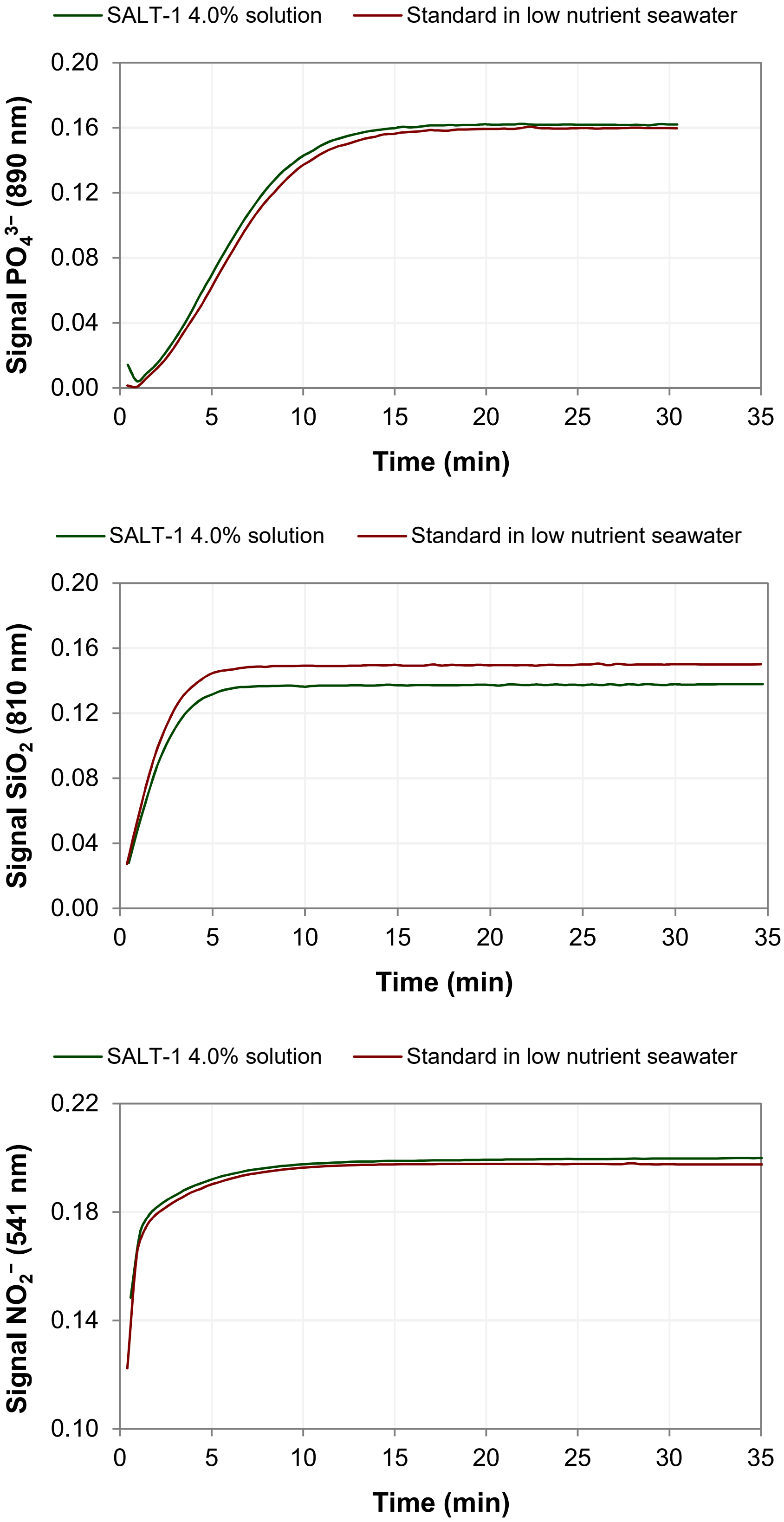

Figure S11. Kinetics of color development: SALT-1 *vs* low nutrient seawater

A 4.0% *w*/*w* SALT-1 solution was spiked with 0.175 µg/g nitrite. Another nutrient solution was prepared in low nutrient seawater to match SALT-1 concentration of phosphate, silicate, and nitrite. After derivatization, the absorbance of the two solutions was recorded against time.

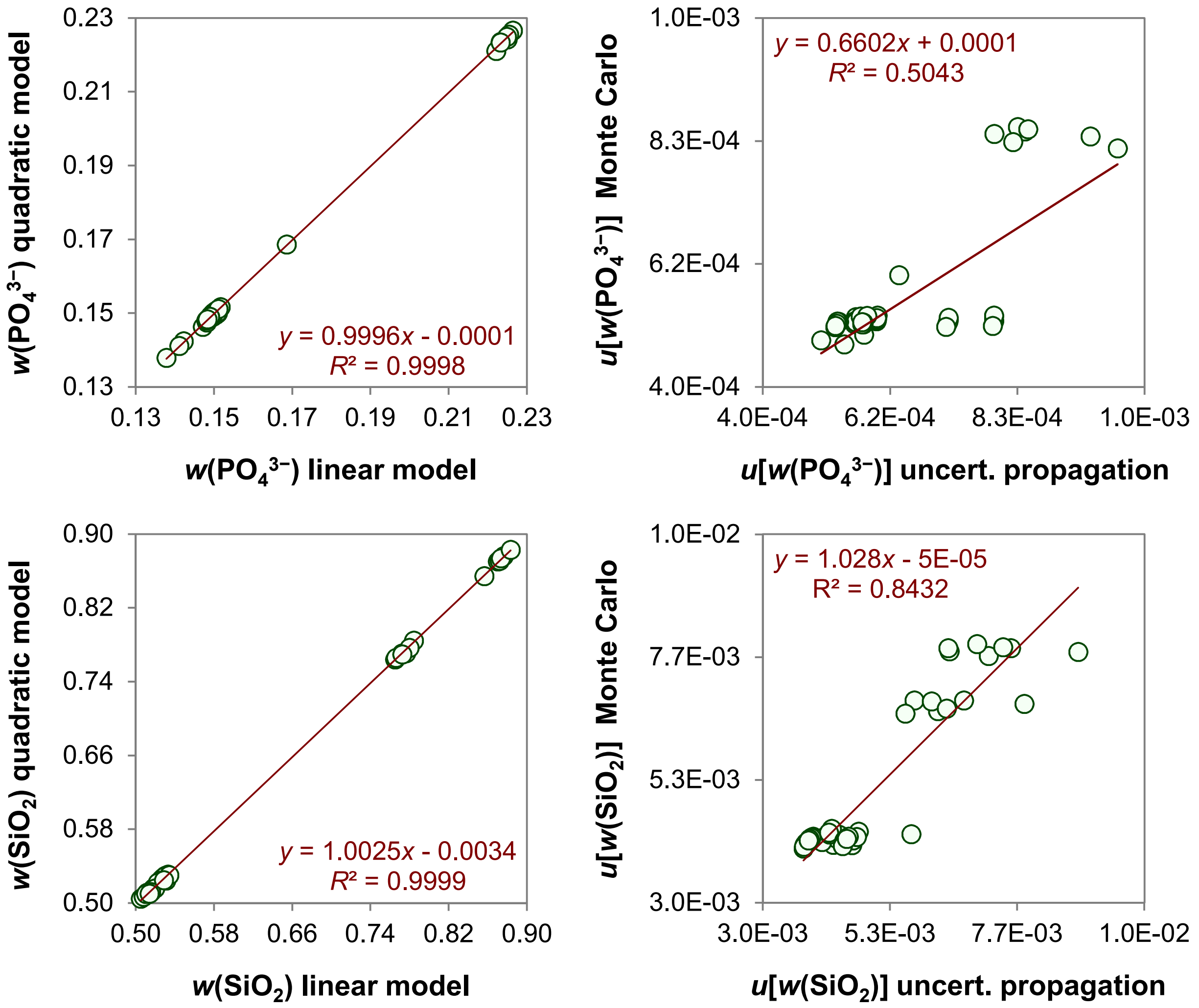

Figure S12. Quantitation of phosphate and silicate by photometry: linear *vs* quadratic model, and error propagation *vs* Monte Carlo

Left: quantitation of phosphate (top) and silicate (bottom) by photometry; comparing quantitative data obtained using either linear or quadratic model for calibration. Right: uncertainty evaluation for phosphate (top) and silicate (bottom); comparing uncertainty generated by propagation (combined standard uncertainty, see Ref. [7] paragraph 5.2, equation 13) and by Monte Carlo method. Details on uncertainty estimation can be found in Paragraphs S1.7 to S1.8 and in the attached Excel file.

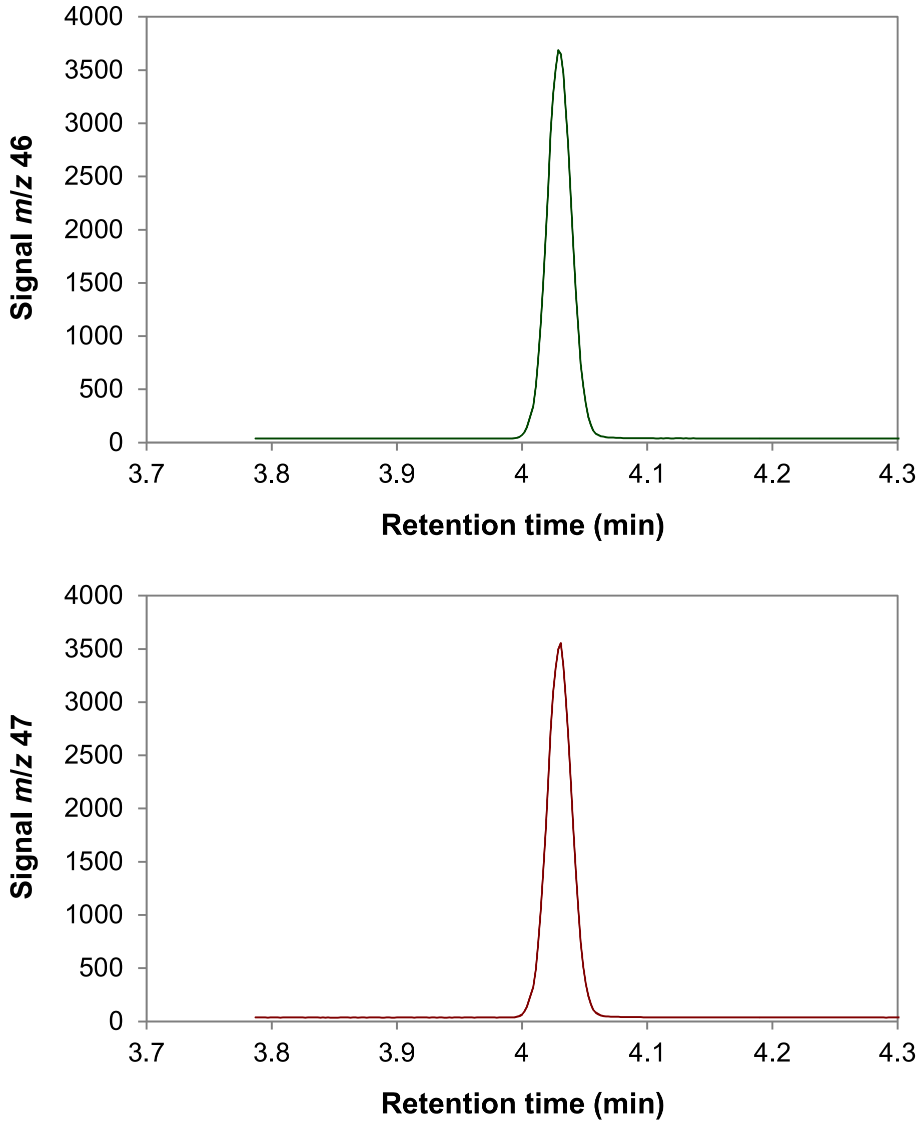

Figure S13. GC–MS nitrate in SALT-1

Headspace GC–MS chromatogram of ethyl nitrate (generated from reaction between NO_3^- and Et_3O^+) in a 4.0% *w*/*w* SALT-1 solution. Top: analytical signal at *m*/*z* 46 (*S*/*N* ratio of 1905, height, peak-to-peak from drift) Bottom: internal standard signal at *m*/*z* 47.

S3. Supporting tables

Table S1. Nutrient stability in 4.0% *w*/*w* solution of SALT-1: effect of pH and time (room temperature)

Day	Phosphate signal (890 nm, with gravimetric correction)		
	pH 8.9	pH 8.0	pH 7.2
1	0.1788	0.1788	0.1791
2	0.1797	0.1789	0.1788
3	0.1775	0.1768	0.1781
8	0.1277	0.1784	0.1783
15	0.0480	0.1781	0.1780
17	0.0415	0.1770	0.1780
22	0.0338	0.1767	0.1775
**Day**	**Silicate signal (810 nm, with gravimetric correction)**		
	**pH 8.9**	**pH 8.0**	**pH 7.2**
1	0.1611	0.1588	0.1544
2	0.1687	0.1680	0.1709
3	0.1750	0.1744	0.1790
8	0.1731	0.1756	0.1799
15	0.1752	0.1773	0.1823
17	0.1764	0.1774	0.1820
22	0.1757	0.1744	0.1785
**Day**	**Nitrate signal (isotope ratio, with gravimetric correction)**		
	**pH 8.9**	**pH 8.0**	**pH 7.2**
1	0.09812	0.09695	0.09721
2	0.09759	0.09779	0.09733
8	0.09822	0.09713	0.09828
15	0.09800	0.09804	0.09904
17	0.09809	0.10087	0.09884
22	0.10014	0.09752	0.09912

Table S2. Solubility of silicate along the reconstitution of SALT-1

**Time**	**Units SALT0295**		**Unit SALT0477**		**Unit SALT0575**	
Hours	Signal	*StDev*	Signal	*StDev*	Signal	*StDev*
3.0	1.0014	*0.0023*	1.0101	*0.0023*	0.9492	*0.0029*
24.4	1.0533	*0.0010*	1.0870	*0.0047*	1.0662	*0.0021*
49.0	1.0535	*0.0034*	1.0835	*0.0021*	1.0559	*0.0036*
73.3	1.0565	*0.0032*	1.0898	*0.0034*	1.0663	*0.0039*
96.8	1.0599	*0.0034*	1.0888	*0.0034*	1.0668	*0.0038*
169.1	1.0640	*0.0025*	1.0895	*0.0023*	1.0633	*0.0024*
192.1	1.0626	*0.0023*	1.0874	*0.0014*	1.0632	*0.0022*
216.8	1.0606	*0.0026*	1.0818	*0.0021*	1.0625	*0.0019*

Signal: average (n = 3) of the silicate signal normalized by the signal of a reference solution prepared by dissolving silicate standard in low nutrient seawater. StDev: standard deviation n = 3. Complete dissolution was observed after 24 hours.

Table S3. Selection of SALT-1 units for the homogeneity study

Unit name	Unit N	Spacing	Bottle ID	Δ Time (min)	Day
SALT0025	0025	24	1	56.92	1
SALT0053	0053	28	2	56.72	1
SALT0111	0111	58	5	3.97	1
SALT0156	0156	45	6	45.95	1
SALT0216	0216	60	9	0.00	2
SALT0282	0282	66	11	28.13	2
SALT0328	0328	46	13	10.20	2
SALT0361	0361	33	14	20.97	2
SALT0445	0445	84	17	33.00	3
SALT0461	0461	16	18	1.28	3
SALT0540	0540	79	20	45.63	3
SALT0595	0595	55	23	14.68	3
SALT0668	0668	73	26	4.17	4
SALT0754	0754	86	29	9.93	4
SALT0820	0820	66	31	24.67	4
SALT0846	0846	26	32	24.57	4

Spacing: number of units produced before the sampling for homogeneity study. *Bottle ID*: before the final bottling, the SALT-1 was portioned in 32 x125 mL Nalgene bottles: the bottle ID column keeps track of the bottle number where the corresponding SALT-1 units was coming from. *Δ Time (min)*: this is the time from when the Bottle ID was opened to when the corresponding SALT-1 unit was produced (monitoring the effect of ambient humidity on the homogeneity). Day: this column keeps track of the day of bottling (the SALT-1 was bottled over four days).

S4. References

1. Zhang J-Z, Ortner PB, Fischer CJ, Arar EJ. Determination of nitrate and nitrite in estuarine and coastal waters by gas segmented continuous flow colorimetric analysis, EPA method 353.4, Revision 2.0, 1997.

2. Grasshoff K, Kremling K, Ehrhardt M. Methods of Seawater Analysis, Third Edition. WILEY-VCH Verlag GmbH; 1999.

3. Zimmermann CF, Keefe CW, Arar EJ. Determination of orthophosphate in estuarine and coastal waters by automated colorimetric analysis, EPA method 365.5, Revision 1.4, 1997.

4. Murphy J, Riley JP. A modified single solution method for the determination of phosphate in natural waters. Anal Chim Acta. 1962;27:31-6. doi: 10.1016/S0003-2670(00)88444-5.

5. Nagul EA, McKelvie ID, Worsfold P, Kolev SD. The molybdenum blue reaction for the determination of orthophosphate revisited: Opening the black box. Anal Chim Acta. 2015;890:60-82. doi: 10.1016/j.aca.2015.07.030.

6. Su-Cheng P, Chung-Cheng Y, Riley JP. Effects of acidity and molybdate concentration on the kinetics of the formation of the phosphoantimonylmolybdenum blue complex. Anal Chim Acta. 1990;229:115-20. doi: 10.1016/S0003-2670(00)85116-8.

7. JCGM 100 (2008) Evaluation of measurement data - Guide to the expression of uncertainty in measurement.

8. JCGM 101 (2008) Evaluation of measurement data - Supplement 1 to the "Guide to the expression of uncertainty in measurement" - Propagation of distributions using a Monte Carlo method.

9. Zhang J-Z, Berberian GA, Arar EJ. Determination of dissolved silicate in estuarine and coastal waters by gas segmented continuous flow colorimetric analysis, EPA method 366.0, Version 1.0, 1997.

10. Campanella B, Onor M, Pagliano E. Rapid determination of nitrate in vegetables by gas chromatography mass spectrometry. Anal Chim Acta. 2017;980:33-40. doi: 10.1016/j.aca.2017.04.053.

11. Pagliano E, Onor M, Pitzalis E, Mester Z, Sturgeon RE, D'Ulivo A. Quantification of nitrite and nitrate in seawater by triethyloxonium tetrafluoroborate derivatization -

Headspace SPME GC-MS. Talanta. 2011;85(5):2511-6. doi: 10.1016/j.talanta.2011.07.098.

12. Pagliano E, Meija J, Sturgeon RE, Mester Z, Dulivo A. Negative chemical ionization GC/MS determination of nitrite and nitrate in seawater using exact matching double spike isotope dilution and derivatization with triethyloxonium tetrafluoroborate. Anal Chem. 2012;84(5):2592-6. doi: 10.1021/ac2030128.

13. Pagliano E, Meija J, Mester Z. High-precision quadruple isotope dilution method for simultaneous determination of nitrite and nitrate in seawater by GCMS after derivatization with triethyloxonium tetrafluoroborate. Anal Chim Acta. 2014;824:36-41. doi: 10.1016/j.aca.2014.03.018.

14. Pagliano E, Meija J, Campanella B, Onor M, Iammarino M, D’Amore T, et al. Certification of nitrate in spinach powder reference material SPIN-1 by high-precision isotope dilution GC–MS. Anal Bioanal Chem. 2019;411(16):3435-45. doi: 10.1007/s00216-019-01803-4.

15. Rienitz O, Jährling R, Noordmann J, Pape C, Röhker K, Vogl J, et al. CCQM-K122 "Anionic impurities and lead in salt solutions". Metrologia. 2020;57(1A):08012. doi: 10.1088/0026-1394/57/1a/08012.

16. Meija J, Pagliano E, Mester Z. Coordinate swapping in standard addition graphs for analytical chemistry: A simplified path for uncertainty calculation in linear and nonlinear plots. Anal Chem. 2014;86(17):8563-7. doi: 10.1021/ac5014749.
